# Supplementary material for: TransLeish: Identification of membrane transporters essential for survival of intracellular Leishmania parasites in a systematic gene deletion screen
Source: Nat Commun. 2025 Jan 2;16:299. doi: 10.1038/s41467-024-55538-7 (PMC11696137; doi:10.1038/s41467-024-55538-7)
Supplement: Supplementary file 1 — Supplementary Information [file 41467_2024_55538_MOESM1_ESM.pdf]

Supplementary Information for

TransLeish: Identification of membrane transporters essential for survival of intracellular Leishmania parasites in a systematic gene deletion screen

Andreia Albuquerque-Wendt, Ciaran McCoy, Rachel Neish, Ulrich Dobramysl, Çağla Alagöz, Tom Beneke, Sally A. Cowley, Kathryn Crouch, Richard J. Wheeler, Jeremy C. Mottram and Eva Gluenz*

*Correspondence: eva.gluenz@unibe.ch

Supplementary Table 1

Details of reagents and resources used in this study.

REAGENT or RESOURCE	SOURCE	IDENTIFIER
Chemicals, peptides, and recombinant proteins		
Medium 199 (M199)	Gibco/ThermoFisher	Cat#31100027
Phosphate buffered saline (PBS tablet)	Sigma-Aldrich/Merck	Cat#P4417
$NaHCO_3$	Sigma-Aldrich/Merck	Cat# S5761
Hemin	Sigma-Aldrich/Merck	Cat#51280
HEPES	Sigma-Aldrich/Merck	Cat#H4034
Fetal bovine serum (FBS)	Gibco	Cat#10270-106
Nourseothricin sulfate	Jena Bioscience	Cat#AB-102
Hygromycin B Gold	InvivoGen	Cat#ant-hg
Puromycin dihydrochloride	InvivoGen	Cat#ant-pr
Blasticidin S hydrochloride	InvivoGen	Cat#ant-bl
mTeSR1	StemCell Technologies	Cat#85850
Geltrex (hESC-qualified)	Invitrogen	Cat#A1413201
EDTA	Invitrogen	Cat#15575-020
TryplE	Invitrogen	Cat#12604013
Rock-inhibitor Y27632	Abcam	Cat#ab120129
Aggrewell 800	StemCell Technologies	Cat#34815
BMP4	Invitrogen	Cat#PHC9534
VEGF	Invitrogen	Cat#PHC9394
SCF	Miltenyi	Cat#130-096-695
M-CSF	Invitrogen	Cat#PHC9501
Interleukin-3	Invitrogen	Cat#PHC0033
Advanced DMEM/F-12	ThermoFisher	Cat#12634010
Human recombinant Insulin solution	Sigma	Cat#I9278-5ML
Nuclease-Free Water	Ambion/Invitrogen	Cat#AM9937
GlutaMax	Thermo Scientific	Cat#35050087
Penicillin-Streptomycin (10,000 U/mL)	ThermoFisher	Cat#15140122
Casy ton	OLS	Cat#5651808
Casy clean	OLS	Cat#5651787
DNA Fragmentase	NEB	Cat#M0348
AMPure XP SPRI Reagent	Beckman	Cat#A63880
Biopterin	Sigma-Aldrich/Merck	Cat#B2517

Adenine hemisulfate (salt)	Sigma-Aldrich/Merck	Cat#A3159
Hoechst 33342	Thermo Scientific	Cat#62249
Critical commercial assays		
DNeasy Blood & Tissue Kit	Qiagen	Cat#69506
Expand™ High Fidelity PCR System	Roche/Merck	Cat#11759078001
High Sensitivity DNA Kit	Agilent	Cat#5067-4626
TruSeq DNA Single Indexes Set A	Illumina	Cat#20015960
TruSeq DNA Single Indexes Set B	Illumina	Cat#20015961
NextSeq 500/550 Mid Output Kit v2.5 (150 Cycles)	Illumina	Cat#20024904
MiSeq Reagent Kit v3 (150-cycle)	Illumina	Cat# MS-102-3001
NEBNext Library Quant Kit	NEB	Cat#E7630
FastGene® Optima HotStart Ready Mix	Lubio Science	Cat#LS29
Qubit™ 1X dsDNA HS Assay Kit	Invitrogen	Cat#Q33231
Experimental models: Cell lines		
hiPSC line SFC840-03-03iPS-OX1-18	[1]	https://ebisc.org/STBCi026-A
hiPSC line SFC841-03-01iPS-OX1-19	[2]	https://ebisc.org/STBCi044-A
hiPSC line SFC856-03-04iPS-OX1-23	[3]	https://ebisc.org/STBCi063-A
Experimental models: Organisms/strains		
L. mexicana: MNYC/BZ/62/M379 Cas9T7M	[4,5]	N/A
L. mexicana: TransLeish mutant cell lines	This paper, Supplementary Data 1	N/A
Mouse: BALB/c	Charles River	028-BalB/cAnNCrl
Oligonucleotides		
Synthetic oligonucleotides for PCR	Synthesized at Thermo Fisher Scientific	N/A
Primer sequences for CRISPR-editing of *L. mexicana*	www.LeishGEdit.net and this paper (Supp. Data 5)	N/A
Primer sequences for *L. mexicana* mutant cell line validation	www.LeishGEdit.net, [5] and this paper (Supp. Data 5)	N/A

G00 sgRNA primer sequence<br>5'-<br>AAAAGCACCGACTCGGTGCCACTTTTTCAAGTTGA<br>TAACGGACTAGCCTTATTTTAACTTGCTATTTCTAG<br>CTCTAAAAC-3'	www.LeishGEdit.net, [4]	N/A
PAGE-purified custom designed p5 and p7 primers with Illumina indexes	Illumina<br>Document #<br>1000000002694 v04<br>January 2018, p. 17 and p.29 and [6]	N/A
Recombinant DNA		
pT-Puro	www.LeishGEdit.net, [4]	N/A
pT-Blast	www.LeishGEdit.net, [4]	N/A
pPLOT-mNG-Blast	www.LeishGEdit.net, [4]	N/A

1 Fernandes, H. J. *et al.* ER Stress and Autophagic Perturbations Lead to Elevated Extracellular alpha-Synuclein in GBA-N370S Parkinson's iPSC-Derived Dopamine Neurons. *Stem Cell Reports* **6**, 342-356, doi:10.1016/j.stemcr.2016.01.013 (2016).

2 Dafinca, R. *et al.* C9orf72 Hexanucleotide Expansions Are Associated with Altered Endoplasmic Reticulum Calcium Homeostasis and Stress Granule Formation in Induced Pluripotent Stem Cell-Derived Neurons from Patients with Amyotrophic Lateral Sclerosis and Frontotemporal Dementia. *Stem Cells* **34**, 2063-2078, doi:10.1002/stem.2388 (2016).

3 Haenseler, W. *et al.* Excess alpha-synuclein compromises phagocytosis in iPSC-derived macrophages. *Scientific reports* **7**, 9003, doi:10.1038/s41598-017-09362-3 (2017).

4 Beneke, T. *et al.* A CRISPR Cas9 high-throughput genome editing toolkit for kinetoplastids. *Royal Society open science* **4**, 170095, doi:10.1098/rsos.170095 (2017).

5 Beneke, T. *et al.* Genome sequence of *Leishmania mexicana* MNYC/BZ/62/M379 expressing Cas9 and T7 RNA polymerase. *Wellcome Open Res* **7**, 294, doi:10.12688/wellcomeopenres.18575.2 (2022).

6 Beneke, T. & Gluenz, E. Bar-seq strategies for the LeishGEdit toolbox. *Molecular and biochemical parasitology* **239**, 111295, doi:10.1016/j.molbiopara.2020.111295 (2020).

Supplementary Figures

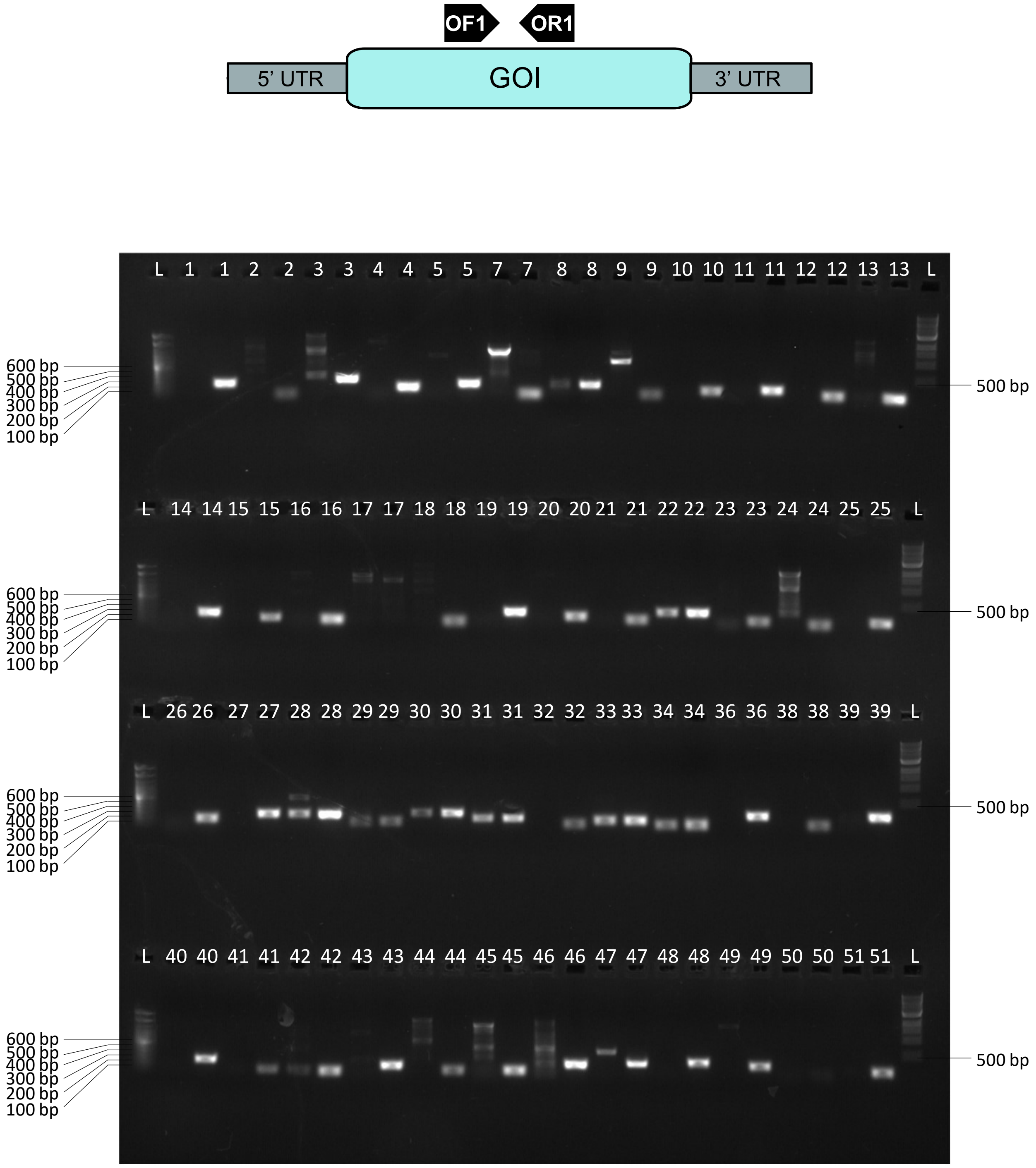

Supp. Fig. 1 (A) Diagnostic PCR amplification of target gene for KO validation

Left lane, Mutant gDNA
Right lane, Parental gDNA (control)

DNA ladders
First lane, 100 bp DNA Ladder, Ref. 15628050, Invitrogen
Last lane, 1 Kb DNA Ladder, Ref. N3232S, NEB

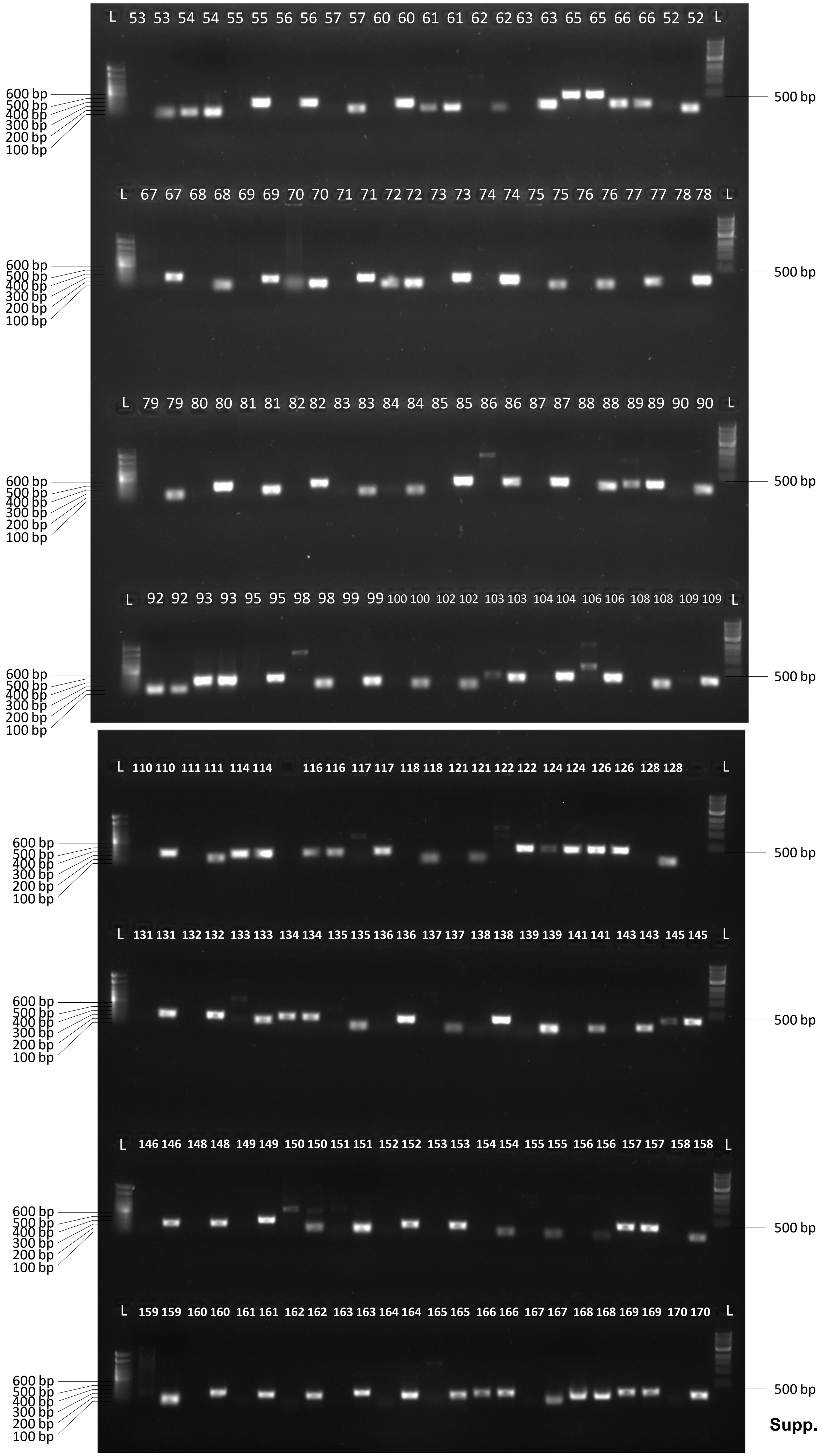
L 53 53 54 54 55 55 56 56 57 57 60 60 61 61 62 62 63 63 65 65 66 66 52 52 L
600 bp
500 bp
400 bp
300 bp
200 bp
100 bp
500 bp
L 67 67 68 68 69 69 70 70 71 71 72 72 73 73 74 74 75 75 76 76 77 77 78 78 L
600 bp
500 bp
400 bp
300 bp
200 bp
100 bp
500 bp
L 79 79 80 80 81 81 82 82 83 83 84 84 85 85 86 86 87 87 88 88 89 89 90 90 L
600 bp
500 bp
400 bp
300 bp
200 bp
100 bp
500 bp
L 92 92 93 93 95 95 98 98 99 99 100 100 102 102 103 103 104 104 106 106 108 108 109 109 L
600 bp
500 bp
400 bp
300 bp
200 bp
100 bp
500 bp
L 110 110 111 111 114 114 116 116 117 117 118 118 121 121 122 122 124 124 126 126 128 128 L
600 bp
500 bp
400 bp
300 bp
200 bp
100 bp
500 bp
L 131 131 132 132 133 133 134 134 135 135 136 136 137 137 138 138 139 139 141 141 143 143 145 145 L
600 bp
500 bp
400 bp
300 bp
200 bp
100 bp
500 bp
L 146 146 148 148 149 149 150 150 151 151 152 152 153 153 154 154 155 155 156 156 157 157 158 158 L
600 bp
500 bp
400 bp
300 bp
200 bp
100 bp
500 bp
L 159 159 160 160 161 161 162 162 163 163 164 164 165 165 166 166 167 167 168 168 169 169 170 170 L
600 bp
500 bp
400 bp
300 bp
200 bp
100 bp
500 bp

Supp. Fig. 1A

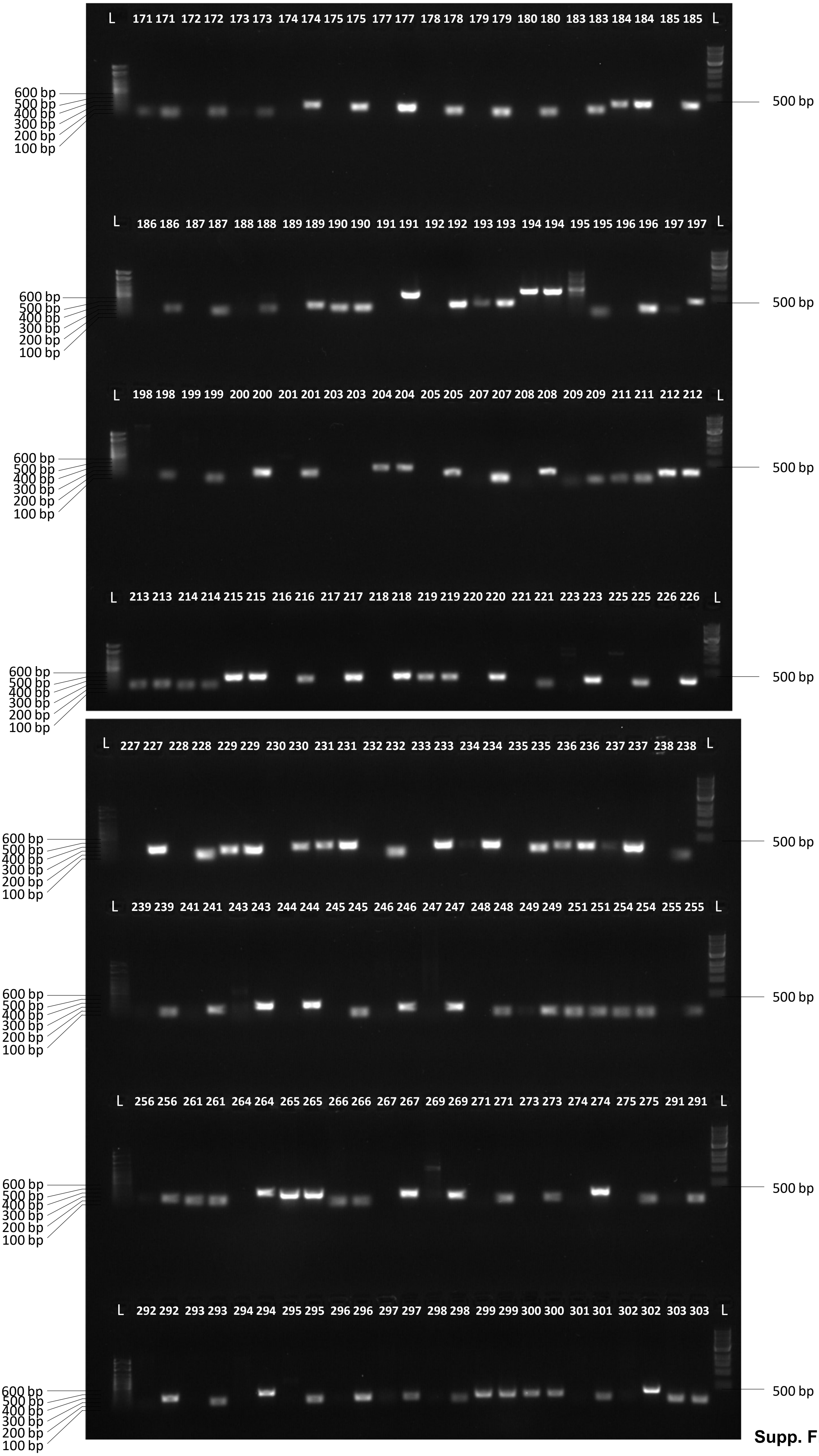

L 171 171 172 172 173 173 174 174 175 175 177 177 178 178 179 179 180 180 183 183 184 184 185 185 L
L 186 186 187 187 188 188 189 189 190 190 191 191 192 192 193 193 194 194 195 195 196 196 197 197 L
L 198 198 199 199 200 200 201 201 203 203 204 204 205 205 207 207 208 208 209 209 211 211 212 212 L
L 213 213 214 214 215 215 216 216 217 217 218 218 219 219 220 220 221 221 223 223 225 225 226 226 L
L 227 227 228 228 229 229 230 230 231 231 232 232 233 233 234 234 235 235 236 236 237 237 238 238 L
L 239 239 241 241 243 243 244 244 245 245 246 246 247 247 248 248 249 249 251 251 254 254 255 255 L
L 256 256 261 261 264 264 265 265 266 266 267 267 269 269 271 271 273 273 274 274 275 275 291 291 L
L 292 292 293 293 294 294 295 295 296 296 297 297 298 298 299 299 300 300 301 301 302 302 303 303 L
600 bp
500 bp
400 bp
300 bp
200 bp
100 bp
500 bp

Supp. Fig. 1A

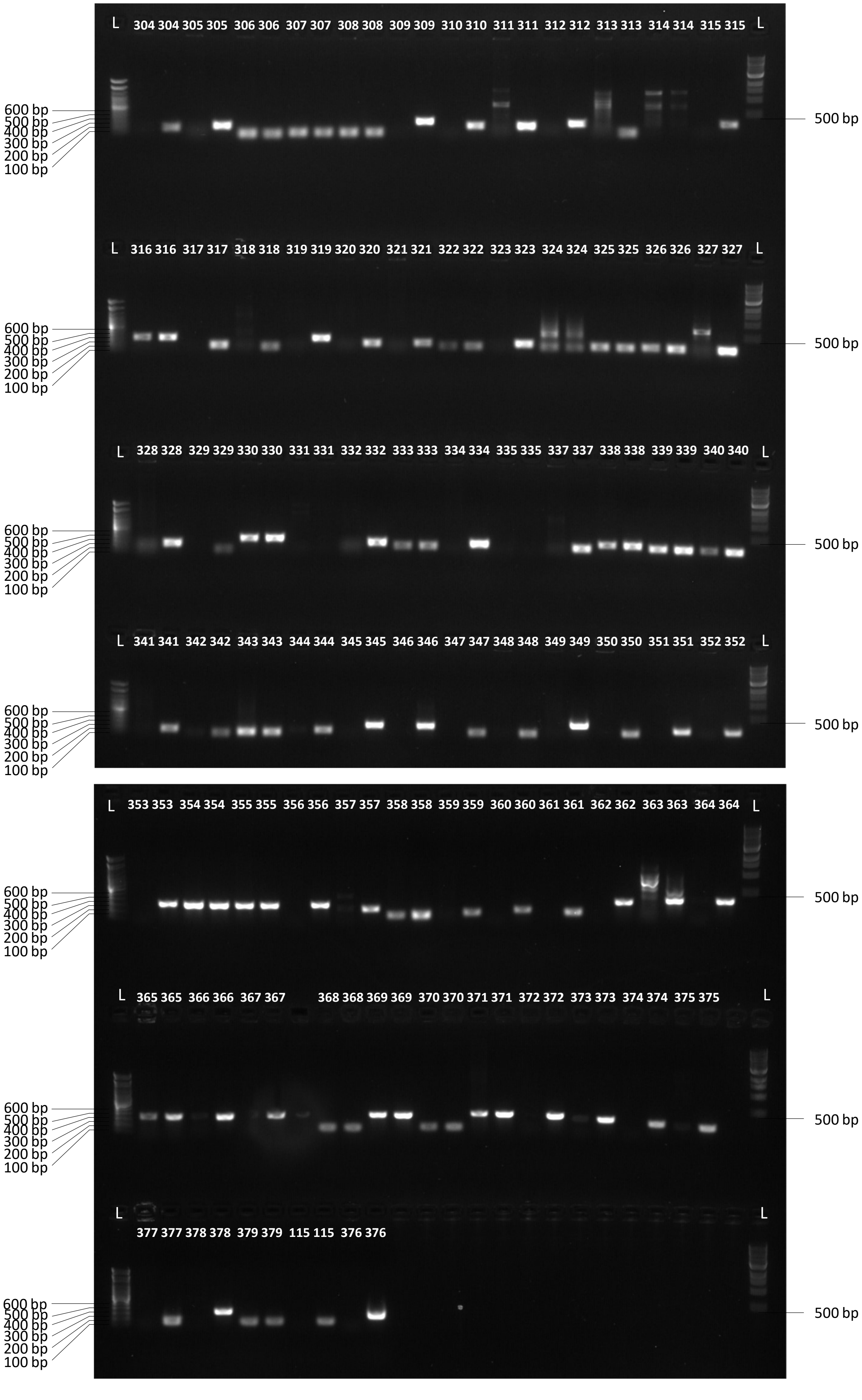
L 304 304 305 305 306 306 307 307 308 308 309 309 310 310 311 311 312 312 313 313 314 314 315 315 L
600 bp
500 bp
400 bp
300 bp
200 bp
100 bp
500 bp
L 316 316 317 317 318 318 319 319 320 320 321 321 322 322 323 323 324 324 325 325 326 326 327 327 L
600 bp
500 bp
400 bp
300 bp
200 bp
100 bp
500 bp
L 328 328 329 329 330 330 331 331 332 332 333 333 334 334 335 335 337 337 338 338 339 339 340 340 L
600 bp
500 bp
400 bp
300 bp
200 bp
100 bp
500 bp
L 341 341 342 342 343 343 344 344 345 345 346 346 347 347 348 348 349 349 350 350 351 351 352 352 L
600 bp
500 bp
400 bp
300 bp
200 bp
100 bp
500 bp
L 353 353 354 354 355 355 356 356 357 357 358 358 359 359 360 360 361 361 362 362 363 363 364 364 L
600 bp
500 bp
400 bp
300 bp
200 bp
100 bp
500 bp
L 365 365 366 366 367 367 368 368 369 369 370 370 371 371 372 372 373 373 374 374 375 375 L
600 bp
500 bp
400 bp
300 bp
200 bp
100 bp
500 bp
L L
377 377 378 378 379 379 115 115 376 376
600 bp
500 bp
400 bp
300 bp
200 bp
100 bp
500 bp

Supp. Fig. 1A

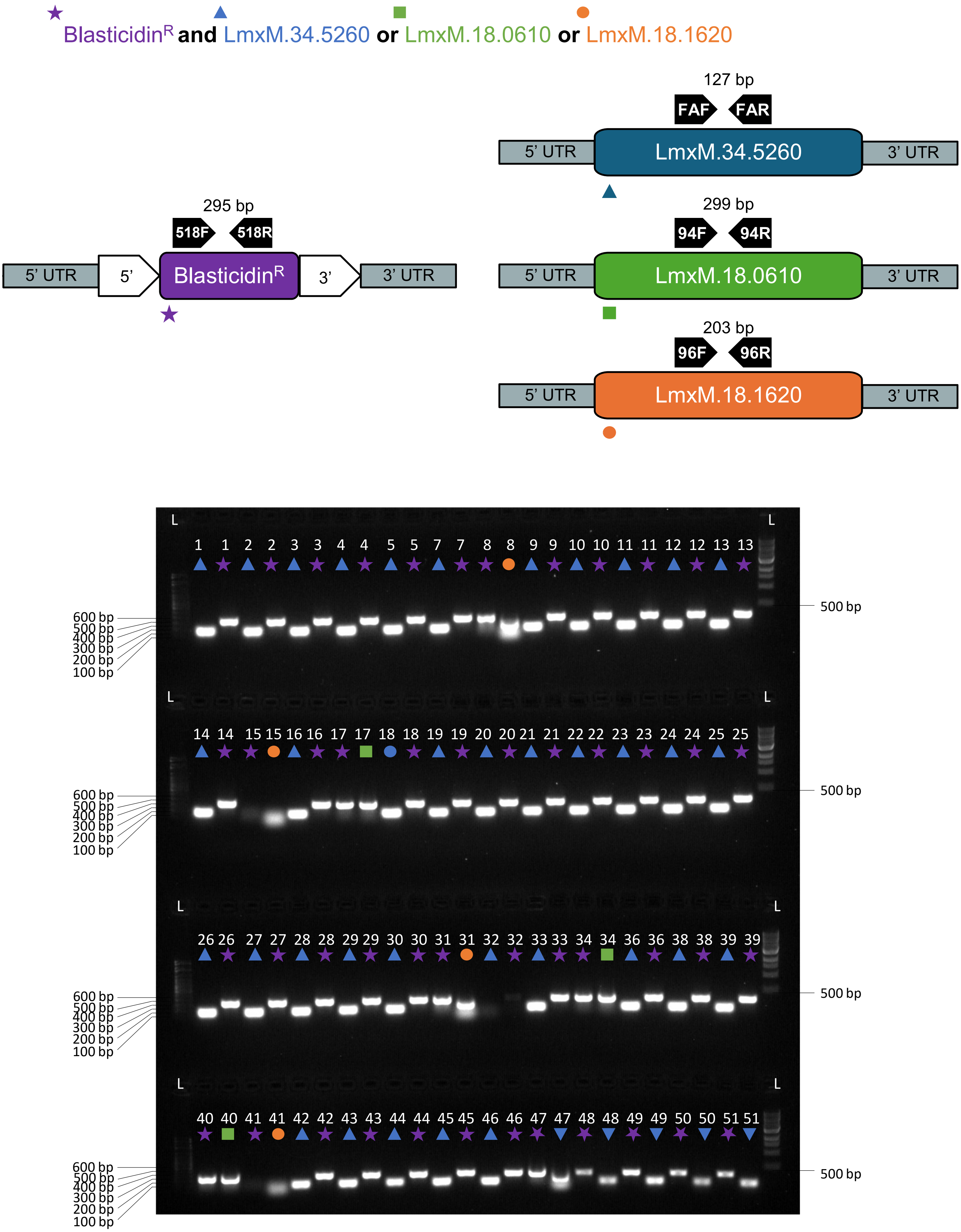

Supp. Fig. 1 (B) Control PCR amplification of a non-target gene from mutant gDNA

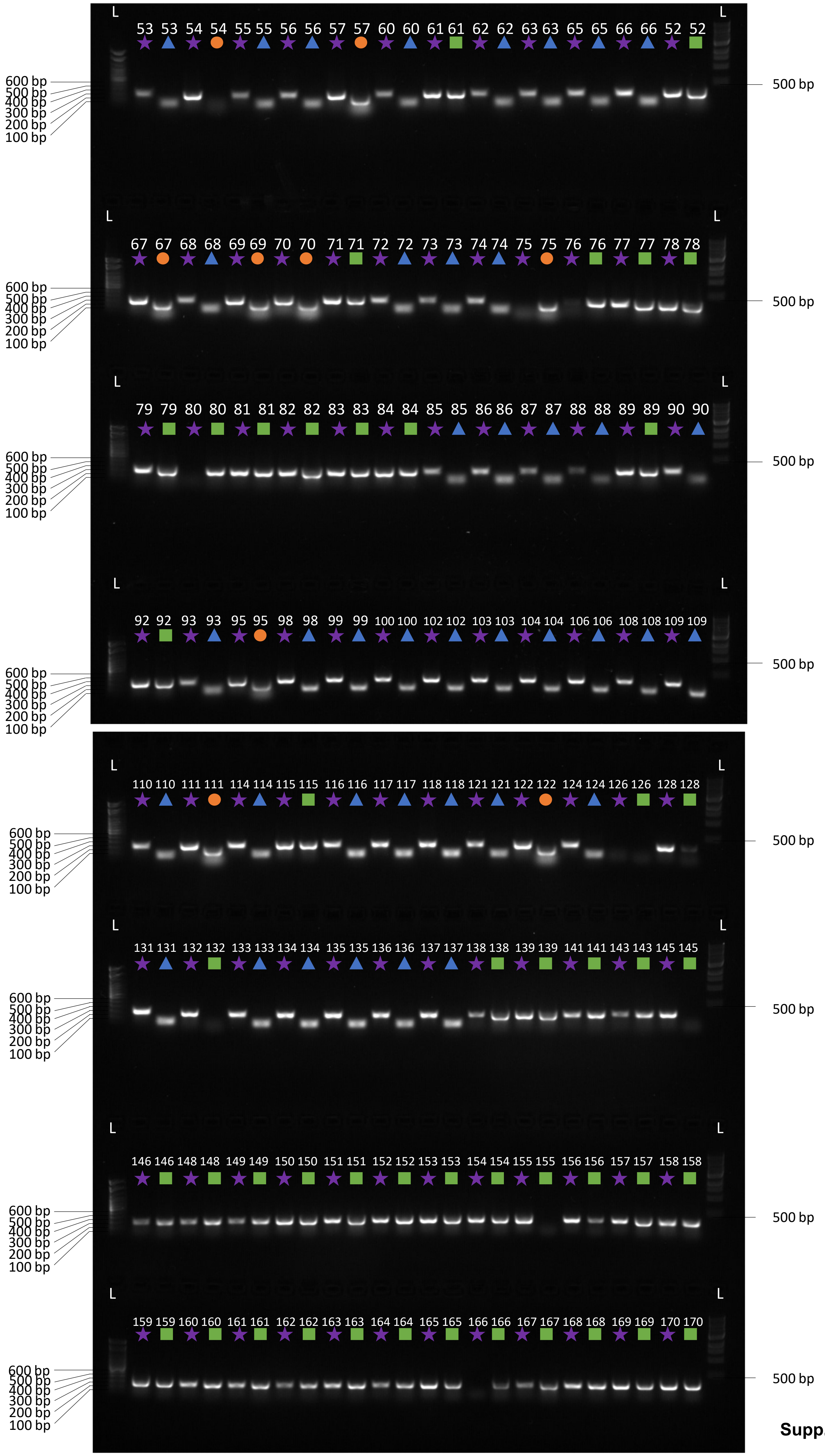
L
53 53 54 54 55 55 56 56 57 57 60 60 61 61 62 62 63 63 65 65 66 66 52 52
600 bp
500 bp
400 bp
300 bp
200 bp
100 bp
500 bp
67 67 68 68 69 69 70 70 71 71 72 72 73 73 74 74 75 75 76 76 77 77 78 78
79 79 80 80 81 81 82 82 83 83 84 84 85 85 86 86 87 87 88 88 89 89 90 90
92 92 93 93 95 95 98 98 99 99 100 100 102 102 103 103 104 104 106 106 108 108 109 109
110 110 111 111 114 114 115 115 116 116 117 117 118 118 121 121 122 122 124 124 126 126 128 128
131 131 132 132 133 133 134 134 135 135 136 136 137 137 138 138 139 139 141 141 143 143 145 145
146 146 148 148 149 149 150 150 151 151 152 152 153 153 154 154 155 155 156 156 157 157 158 158
159 159 160 160 161 161 162 162 163 163 164 164 165 165 166 166 167 167 168 168 169 169 170 170

Supp. Fig. 1B

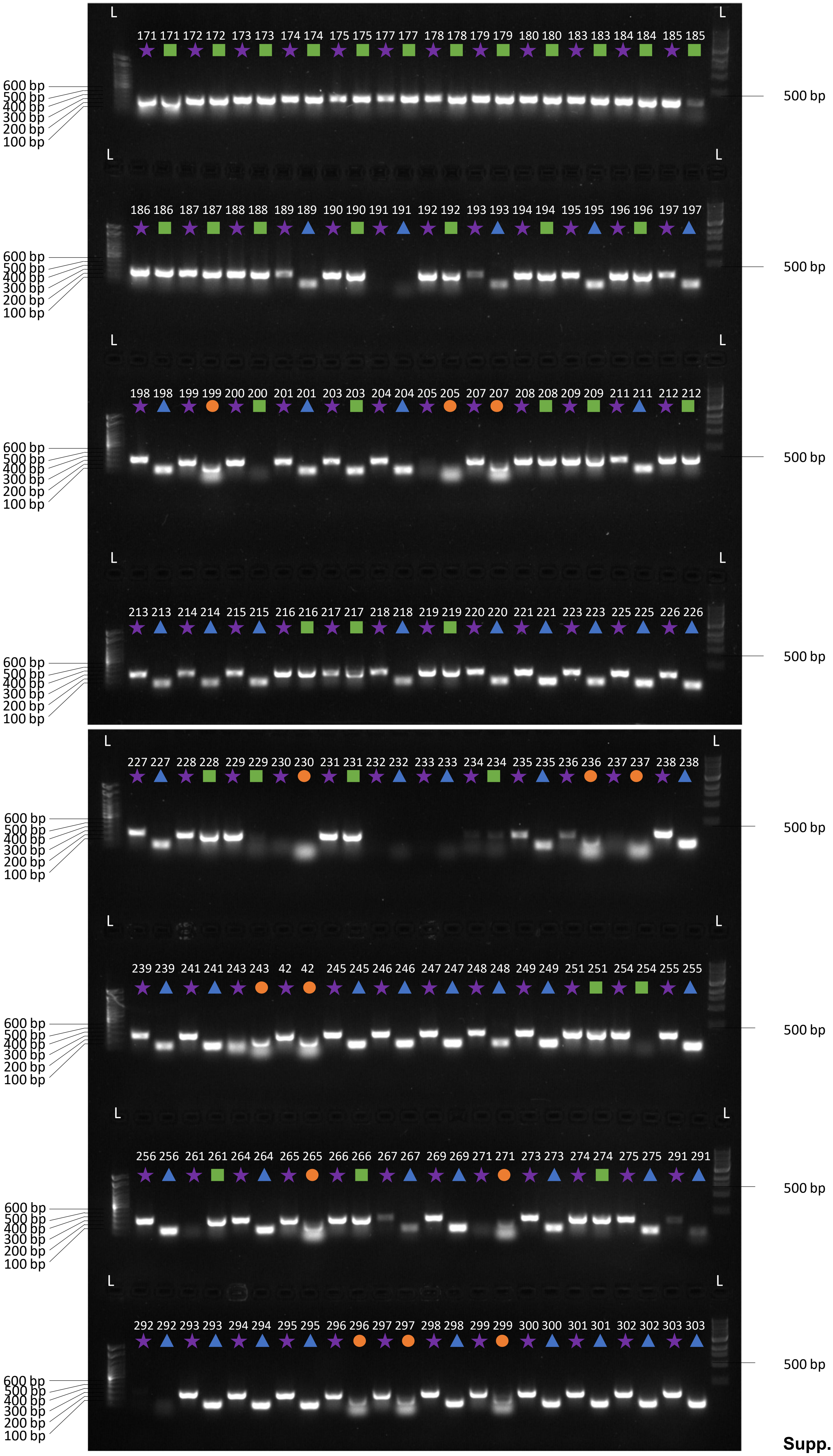
L
171 171 172 172 173 173 174 174 175 175 177 177 178 178 179 179 180 180 183 183 184 184 185 185
600 bp
500 bp
400 bp
300 bp
200 bp
100 bp
500 bp
186 186 187 187 188 188 189 189 190 190 191 191 192 192 193 193 194 194 195 195 196 196 197 197
198 198 199 199 200 200 201 201 203 203 204 204 205 205 207 207 208 208 209 209 211 211 212 212
213 213 214 214 215 215 216 216 217 217 218 218 219 219 220 220 221 221 223 223 225 225 226 226
227 227 228 228 229 229 230 230 231 231 232 232 233 233 234 234 235 235 236 236 237 237 238 238
239 239 241 241 243 243 42 42 245 245 246 246 247 247 248 248 249 249 251 251 254 254 255 255
256 256 261 261 264 264 265 265 266 266 267 267 269 269 271 271 273 273 274 274 275 275 291 291
292 292 293 293 294 294 295 295 296 296 297 297 298 298 299 299 300 300 301 301 302 302 303 303

Supp. Fig. 1B

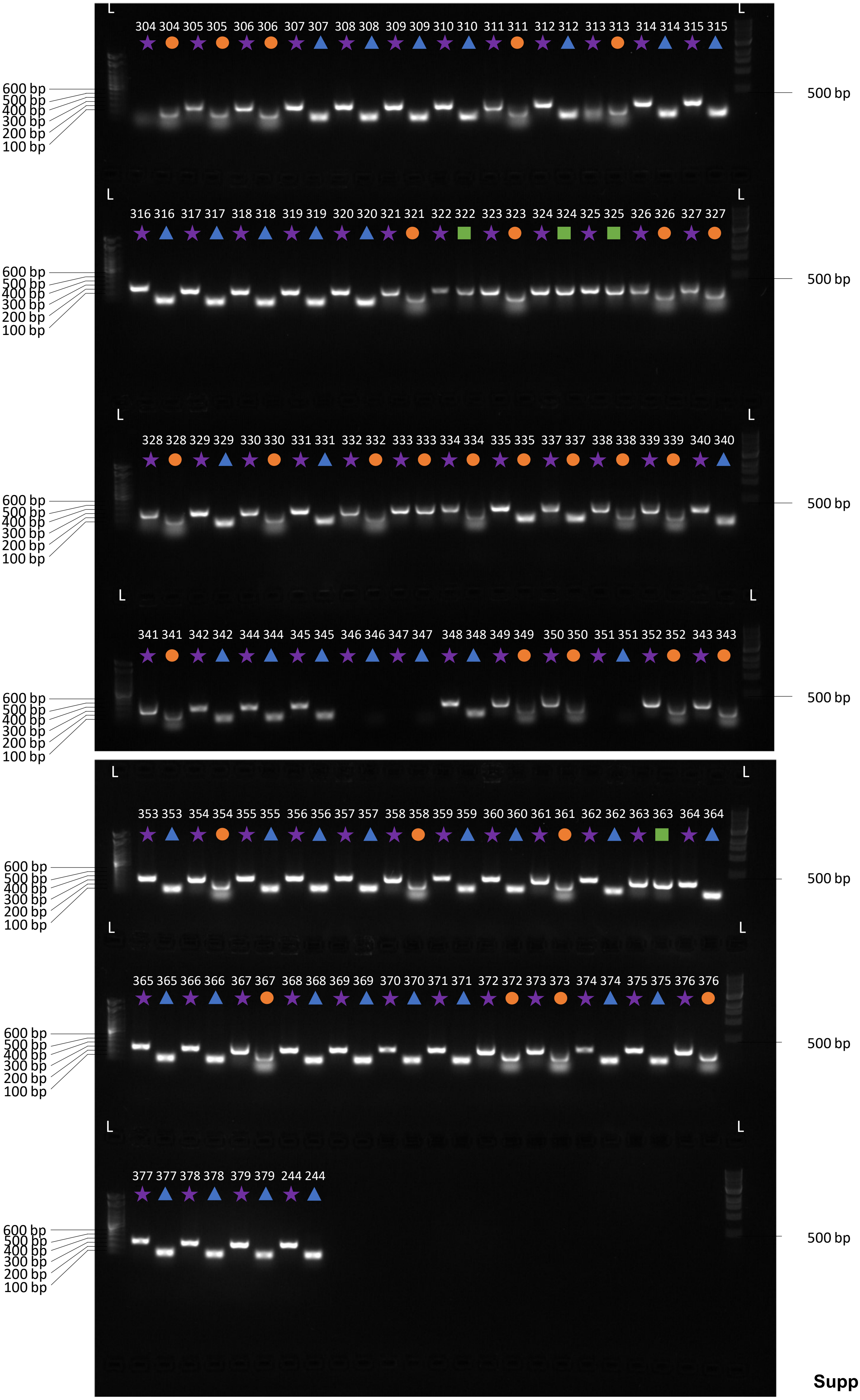
L
304 304 305 305 306 306 307 307 308 308 309 309 310 310 311 311 312 312 313 313 314 314 315 315
600 bp
500 bp
400 bp
300 bp
200 bp
100 bp
500 bp
316 316 317 317 318 318 319 319 320 320 321 321 322 322 323 323 324 324 325 325 326 326 327 327
328 328 329 329 330 330 331 331 332 332 333 333 334 334 335 335 337 337 338 338 339 339 340 340
341 341 342 342 344 344 345 345 346 346 347 347 348 348 349 349 350 350 351 351 352 352 343 343
353 353 354 354 355 355 356 356 357 357 358 358 359 359 360 360 361 361 362 362 363 363 364 364
365 365 366 366 367 367 368 368 369 369 370 370 371 371 372 372 373 373 374 374 375 375 376 376
377 377 378 378 379 379 244 244

Supp. Fig. 1B

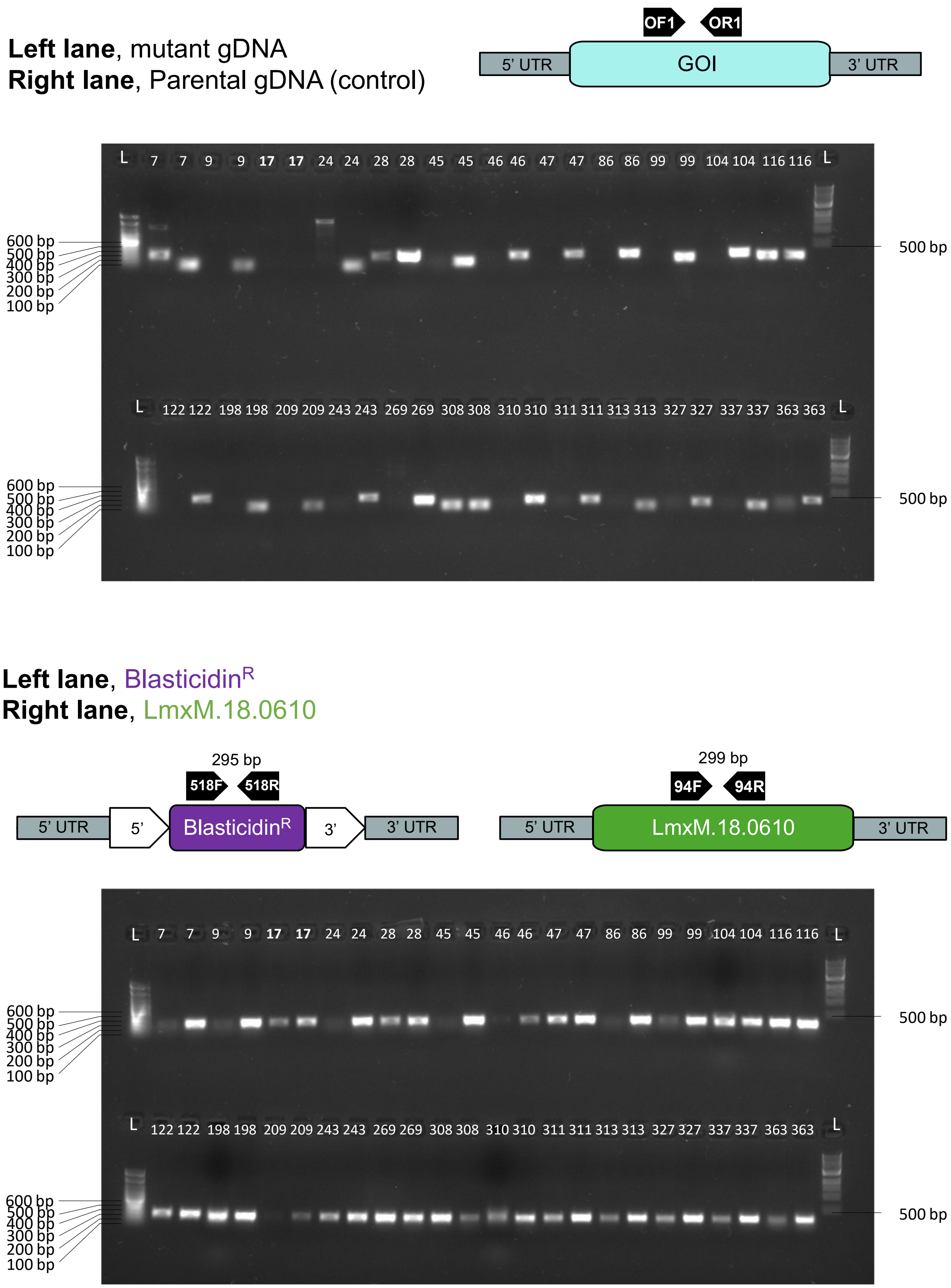

Supp. Fig. 1 (C) Repeats of diagnostic PCR for selected mutants as in A and B, but using a higher annealing temperature of 60ºC. Top, target gene ORF amplification, bottom, control PCR amplification of non-target gene from mutant gDNA.

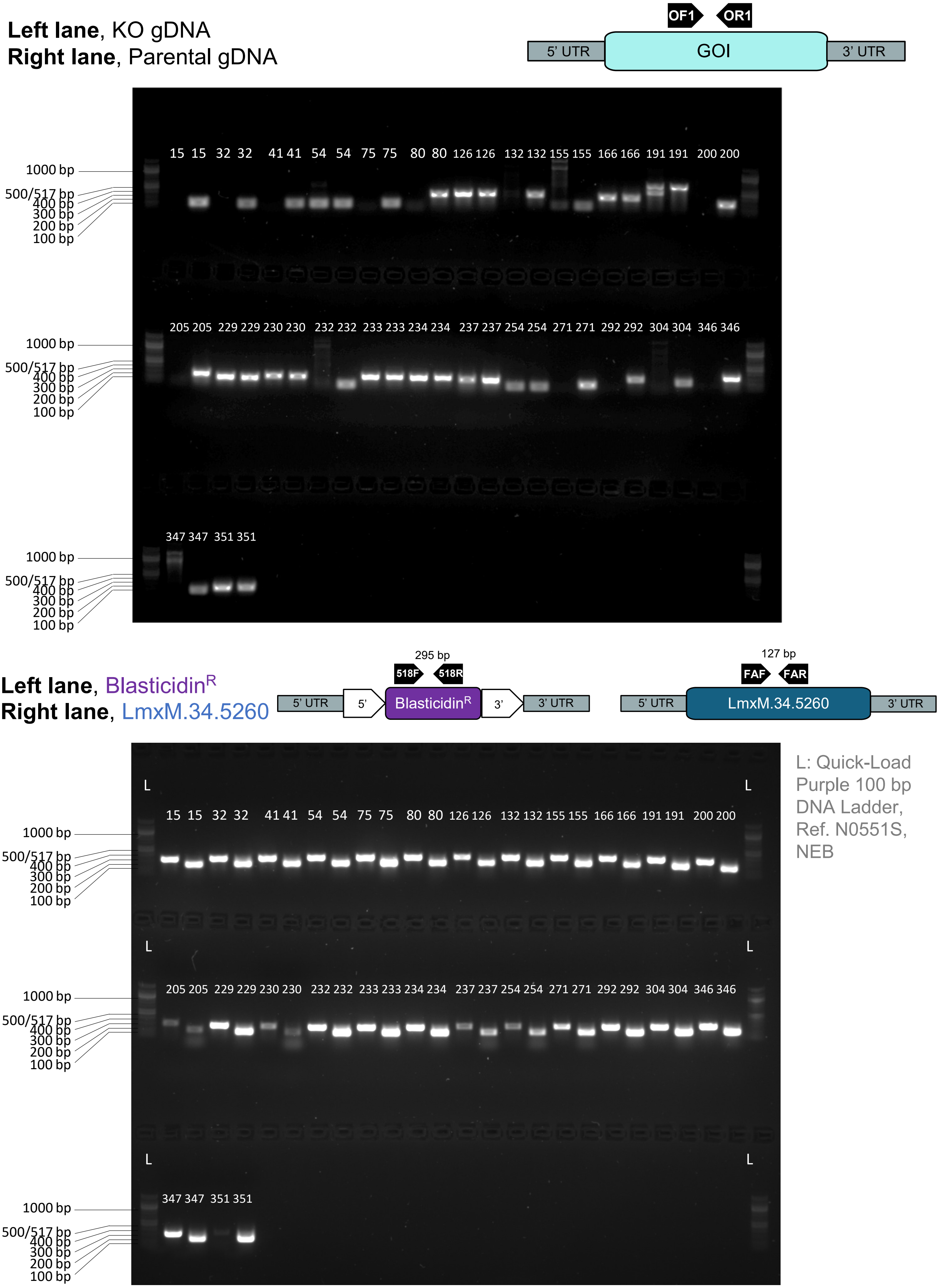

Supp. Fig. 1 (D) Repeats of diagnostic PCR for selected mutants where results from the first PCR were unclear, using the same conditions as in (A) and (B). Top, target gene ORF amplification, bottom, control PCR amplification of non-target gene from mutant gDNA.

Supplementary Figure 1. Diagnostic PCR analysis to determine gene deletion status.
(A) Diagnostic PCR amplification of target gene for knockout validation. The open reading frame of the gene of interest (GOI) was amplified from gDNA with a forward (OF) and reverse (OR) primer pair and products assessed on an agarose gel. Two samples were assessed for each GOI: amplification from *L. mex* Cas9 T7 parental gDNA and amplification from gDNA of mutants that survived transfection and drug selection. Numbers above gel lanes refer to the mutant numbers, as listed in Supplementary Data 2. (B) Control PCR amplification of a non-target gene from mutant gDNA. From each mutant gDNA sample, a fragment of the Blasticidin resistance cassette was amplified with primers BF1 and BR1 (lanes marked with a purple star symbol) and of a second gene as depicted in the schematic above the gel image. Gel lanes are numbered as in A. (C) Repeats of diagnostic PCR for selected mutants as in A and B, but using a higher annealing temperature of 60ºC. (D) Repeats of diagnostic PCR for selected mutants where results from the first PCR were unclear, using the same conditions as in (A) and (B). See Supplementary Data 1 for a summary of diagnostic PCR results.

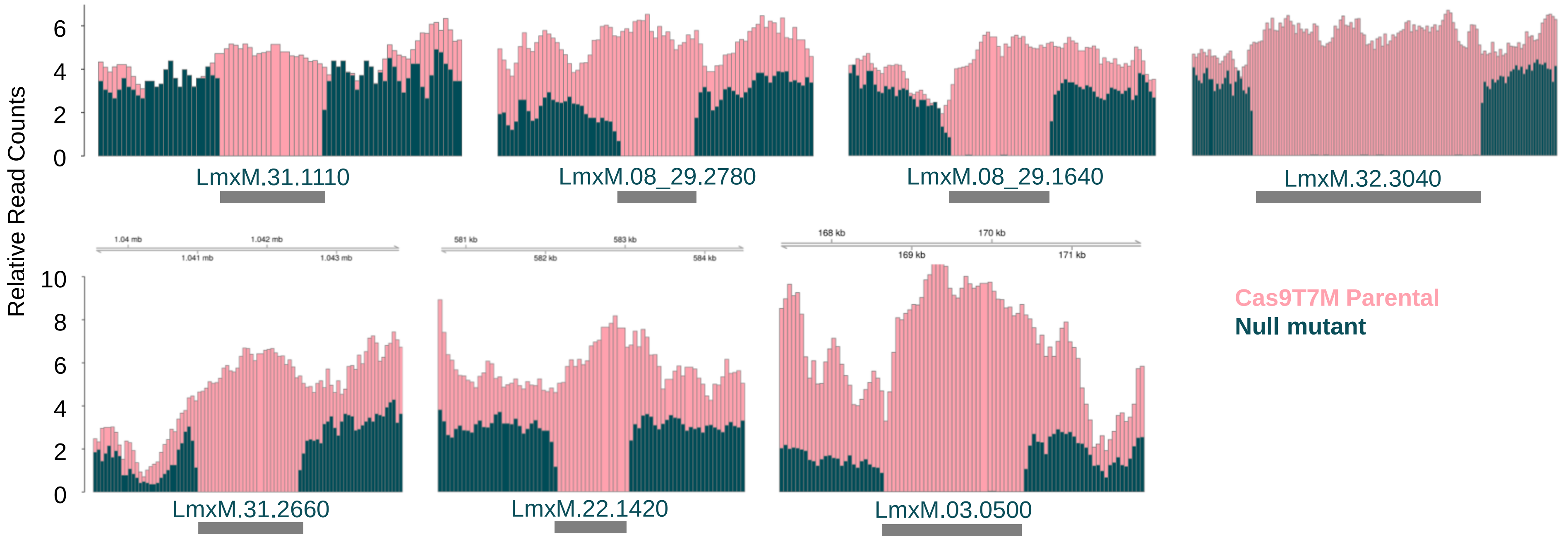

Supplementary Figure 2. Assessment of gene deletion by whole genome sequencing of selected mutants.

Whole genome sequencing was carried out for puromycin and blasticidin resistant mutant lines Δ*LmxM.03.500*, Δ*LmxM.08_29.2780*, Δ*LmxM.08_29.1640*, Δ*LmxM.22.1420*, Δ*LmxM.32.3040*, Δ*LmxM.31.2660* and Δ*LmxM.31.1110* and the *L. mex* Cas9T7 parental cell line. The plots show the relative read counts across the target gene ORF (marked by grey bar) plus 1.5kb of flanking sequences upstream and downstream of the target gene (CPM-normalised coverage in 50bp bins). Reads from the mutant line are coloured green and the reads from the parental line are coloured pink.

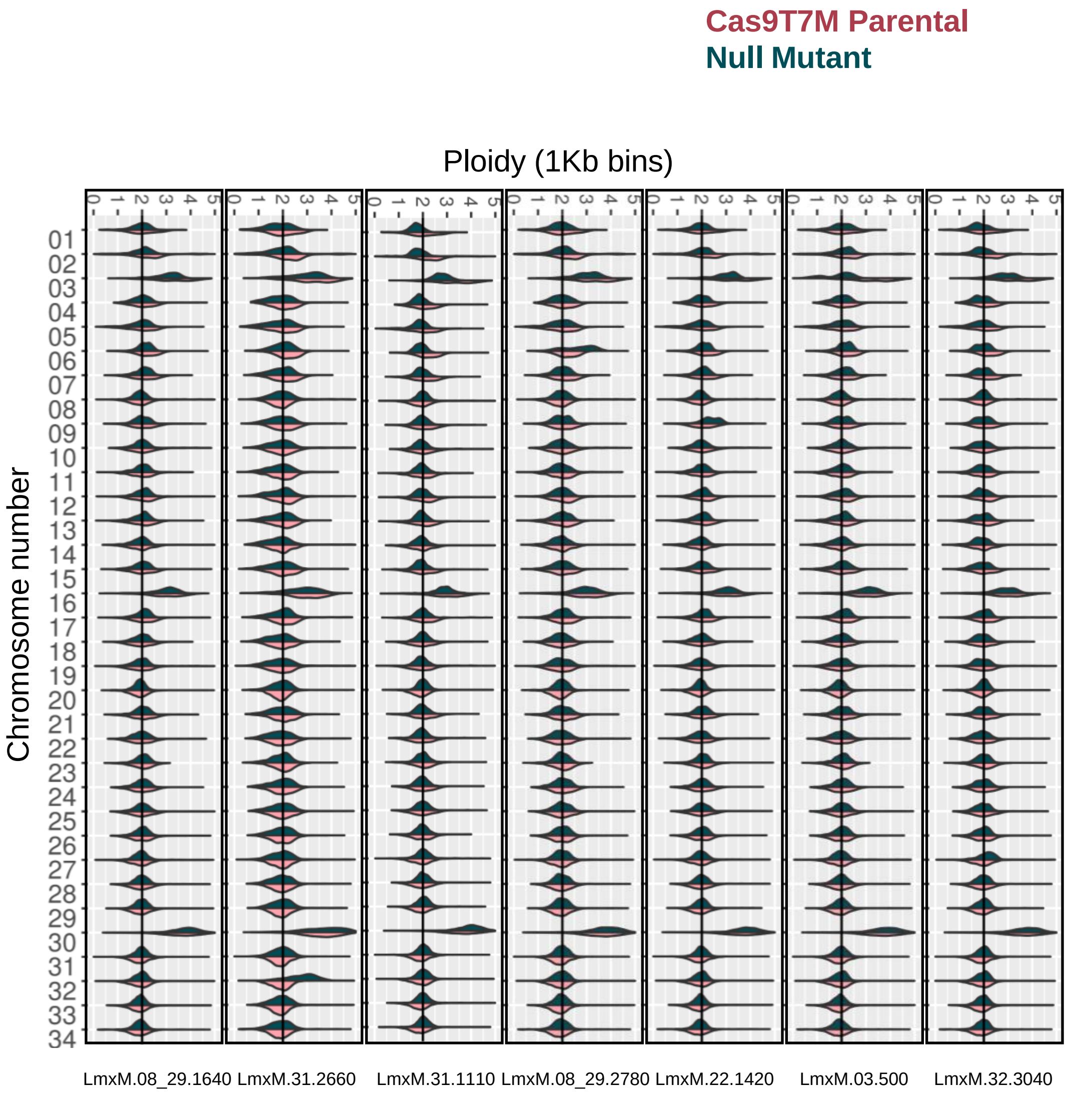

Supplementary Figure 3. Analysis of chromosome ploidy in selected mutants.

Whole genome sequencing read coverage for each chromosome was scaled to ploidy by dividing the coverage value for each bin by median coverage/2. The distribution of scaled coverage is plotted for each chromosome for each mutant line (green) and the parental line (pink). Mutant Δ*LmxM.31.2660*, showed an increase of chromosome 32 from 2n to 3n, mutant Δ*LmxM.08_29.2780*, showed an increase of chromosome 6 from 2n to 3n and in mutant Δ*LmxM.03.0500*, chromosome 3 decreased from 3n to 2n. A shift towards lower copy numbers for chromosome 3 was also seen in the knockout lines Δ*LmxM.08_29.1640*, Δ*LmxM.31.1110*, Δ*LmxM.08_29.2780*, Δ*LmxM.22.1420* and Δ*LmxM.32.3040*, when compared to the parental.

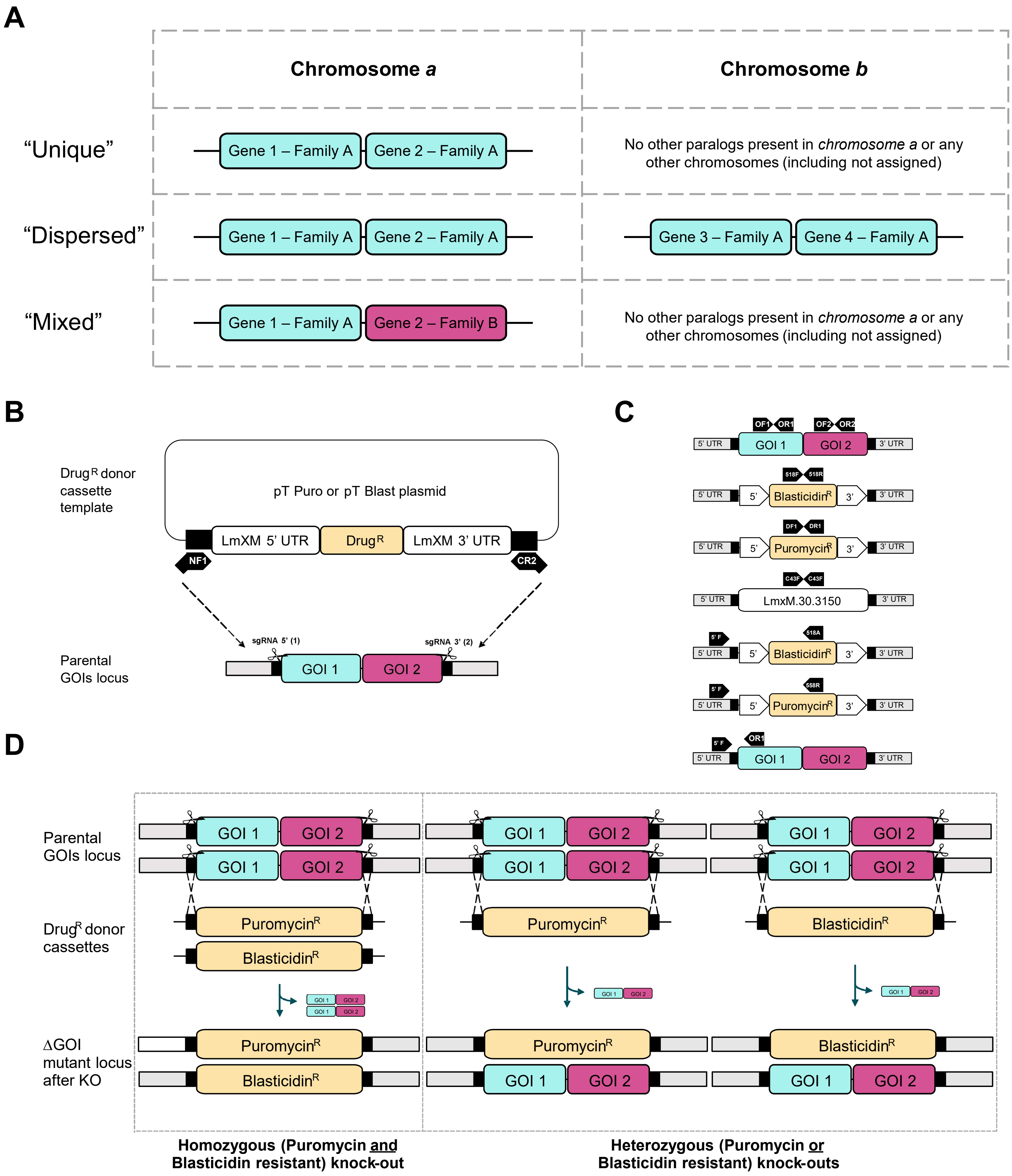
A
Chromosome a
Chromosome b
"Unique"
Gene 1 – Family A
Gene 2 – Family A
No other paralogs present in chromosome a or any other chromosomes (including not assigned)
"Dispersed"
Gene 1 – Family A
Gene 2 – Family A
Gene 3 – Family A
Gene 4 – Family A
"Mixed"
Gene 1 – Family A
Gene 2 – Family B
No other paralogs present in chromosome a or any other chromosomes (including not assigned)
B
Drug R donor cassette template
pT Puro or pT Blast plasmid
LmXM 5' UTR
Drug R
LmXM 3' UTR
NF1
CR2
sgRNA 5' (1)
sgRNA 3' (2)
Parental GOIs locus
GOI 1
GOI 2
C
OF1
OR1
OF2
OR2
5' UTR
GOI 1
GOI 2
3' UTR
518F
518R
5'
Blasticidin R
3'
DF1
DR1
Puromycin R
C43F
LmxM.30.3150
5' F
518A
558R
D
Parental GOIs locus
Drug R donor cassettes
Puromycin R
Blasticidin R
ΔGOI mutant locus after KO
Homozygous (Puromycin and Blasticidin resistant) knock-out
Heterozygous (Puromycin or Blasticidin resistant) knock-outs

Supp. Fig. 4

Supplementary Figure 4. Strategy for tandem array knockouts.

(A) Cartoon illustrating the possible arrangements of transporter-encoding genes in the genome in tandem arrays. Genes fitting into the category ñUniqueò, where a tandem array contained genes from the same family, and no paralogs were detected at other genomic loci, the array was targeted for deletion. (B) CRISPR-Cas9 strategy for array replacement using drug-selectable donor DNA cassettes, with homology arms targeting 30 nt upstream of the first gene in the array and 30 nt downstream of the end of the last gene in the array. (C) Strategy for diagnostic PCRs to determine whether the drug resistance cassettes are linked to the target gene locus in heterozygous knockouts. From top to bottom, the diagnostic PCRs amplified the following: target genes from the array, blasticidin resistance gene from the donor DNA cassette, a gene (*LmxM.30.3150*) from a different genomic locus, blasticidin resistance gene that was inserted in the target array locus, puromycin resistance gene that was inserted in the target array locus, 5ôend of the target gene from the target locus. Black arrows indicate PCR primers: OF and OR primers are specific to the target genes, 5ô F primers bind to the genomic locus upstream of the donor DNA insertion site. (D) Schematic showing modified targeted loci and primers used for diagnostic PCRs (black arrows). The left side of the panel shows a successful array knockout with two drug resistance cassettes. The right side shows the result of targeting the locus with one cassette only (either Blasticidin^R or Puromycin^R), which results in heterozygous mutants.

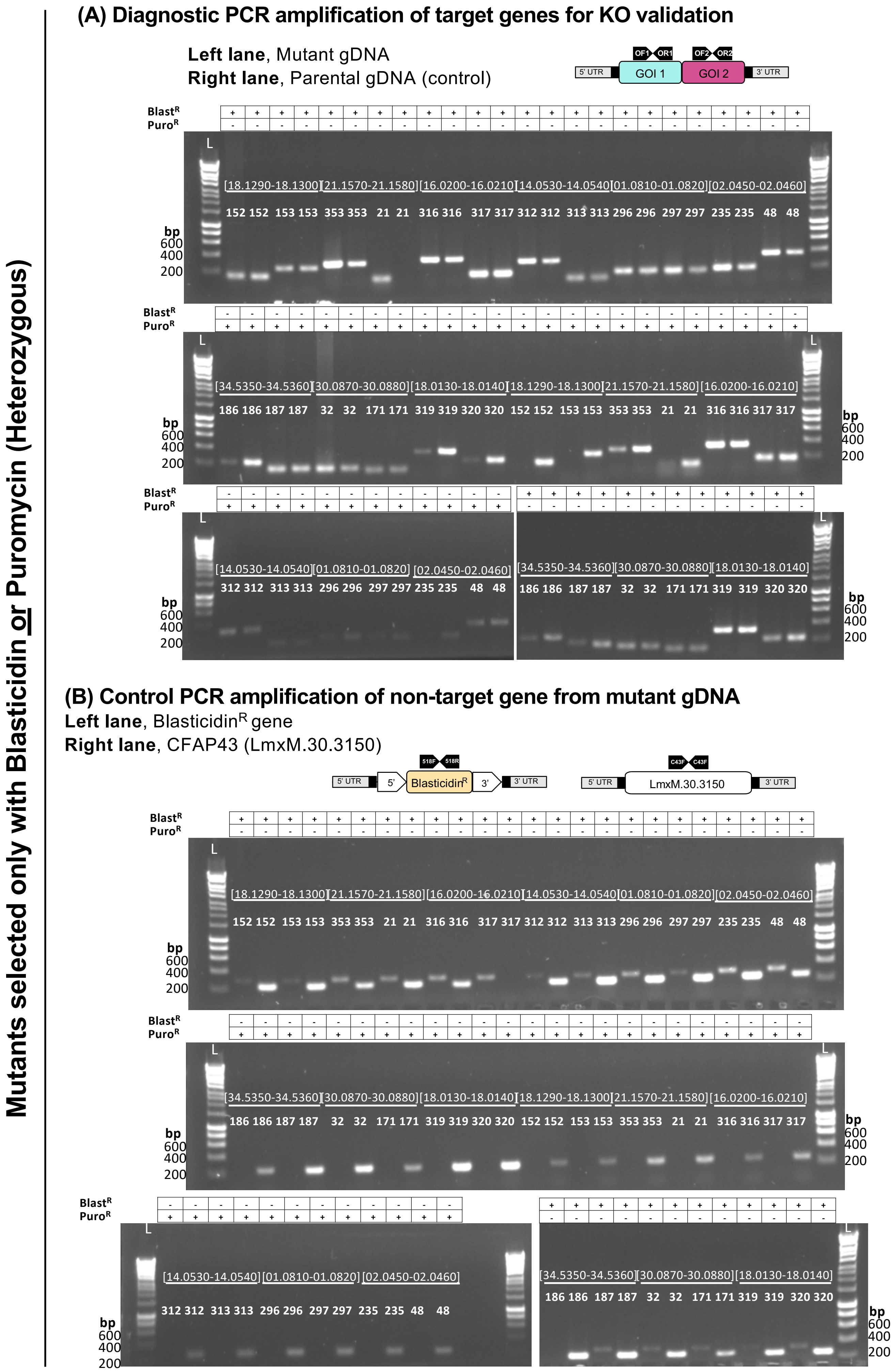
Mutants selected only with Blasticidin or Puromycin (Heterozygous)
(A) Diagnostic PCR amplification of target genes for KO validation
Left lane, Mutant gDNA
Right lane, Parental gDNA (control)
OF1 OR1 OF2 OR2
5' UTR GOI 1 GOI 2 3' UTR
BlastR
PuroR
L
[18.1290-18.1300] [21.1570-21.1580] [16.0200-16.0210] [14.0530-14.0540] [01.0810-01.0820] [02.0450-02.0460]
152 152 153 153 353 353 21 21 316 316 317 317 312 312 313 313 296 296 297 297 235 235 48 48
[34.5350-34.5360] [30.0870-30.0880] [18.0130-18.0140] [18.1290-18.1300] [21.1570-21.1580] [16.0200-16.0210]
186 186 187 187 32 32 171 171 319 319 320 320 152 152 153 153 353 353 21 21 316 316 317 317
[14.0530-14.0540] [01.0810-01.0820] [02.0450-02.0460]
312 312 313 313 296 296 297 297 235 235 48 48
[34.5350-34.5360] [30.0870-30.0880] [18.0130-18.0140]
186 186 187 187 32 32 171 171 319 319 320 320
bp
600
400
200
(B) Control PCR amplification of non-target gene from mutant gDNA
Left lane, BlasticidinR gene
Right lane, CFAP43 (LmxM.30.3150)
518F 518R
5' UTR 5' BlasticidinR 3' 3' UTR
C43F C43F
5' UTR LmxM.30.3150 3' UTR

Supp. Fig. 5

Mutants selected only with Blasticidin or Puromycin (Heterozygous)

(C) Control PCR amplification of 5' UTR and drug resistance gene from mutant gDNA

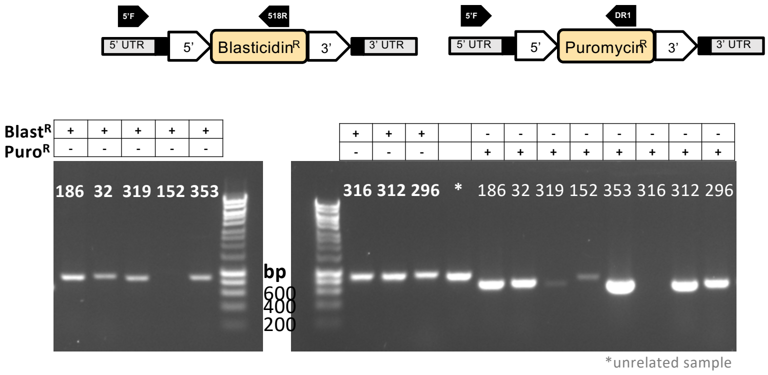

(D) Control PCR amplification of 5' UTR and target gene from mutant gDNA

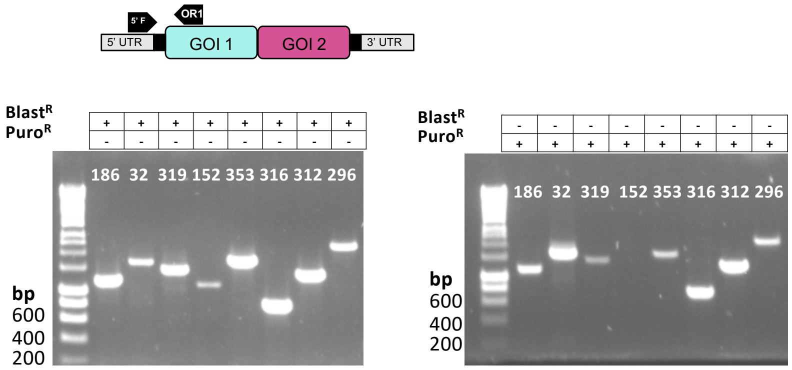

Mutants selected with Blasticidin and Puromycin

(E) Diagnostic PCR amplification of target genes for KO validation

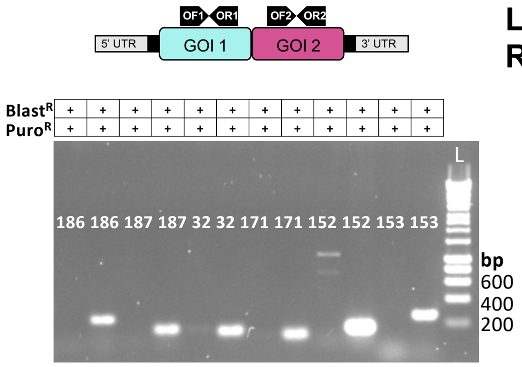

Left lane, mutant gDNA

Right lane, Parental gDNA (control)

(F) Control PCR amplification of the Blasticidin resistance gene from mutant gDNA

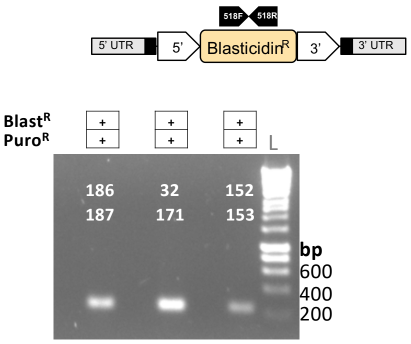

Blasticidin^R gene

Supp. Fig. 5

Supplementary Figure 5. Results of tandem array knockouts.

(A-D) Diagnostic PCRs of heterozygous (Puromycin or Blasticidin resistant) array knockouts, using the strategy shown in Supplementary Figure 4C. Numbers above gel lanes refer to the mutant numbers, as listed in Supplementary Data 1. (A) Diagnostic PCR amplification of each gene from the targeted arrays for knockout validation. Two DNA samples were tested for each GOI: the gDNA of mutants that survived transfection and drug selection (left lane) and *L. mex* Cas9 T7 parental gDNA (right lane). (B) Control PCR amplification of non-target gene from mutant gDNA. From each mutant gDNA sample, a fragment of the Blasticidin resistance cassette was amplified with primers BF1 and BR1 and of a second control gene as depicted in the schematic above the gel image. Gel lanes are numbered as in A. (C) Control PCR amplification of 5ôUTR and drug resistance gene from mutant gDNA. This diagnostic PCR amplifies a fragment that links the 5ôUTR of the GOI to the integrated drug selection marker thus testing for integration of the donor DNA cassette into the target locus. Gel lanes are numbered as in A. (D) Control PCR amplification of 5ô UTR and target gene from mutant gDNA. For single drug resistant array mutants, this diagnostic PCR tests for the retention of the first GOI in the array in the targeted locus. Gel lanes are numbered as in A. (E-F) Diagnostic PCRs of homozygous Puromycin and Blasticidin resistant array knockouts, using the strategy shown in Supplementary Figure 4C. (E) Diagnostic PCR amplification of target genes for knockout validation. Gel lanes are numbered as in A. (F) Control PCR amplification of the Blasticidin resistance gene from mutant gDNA. The uncropped source images are provided at the end of the Supplementary Figures.

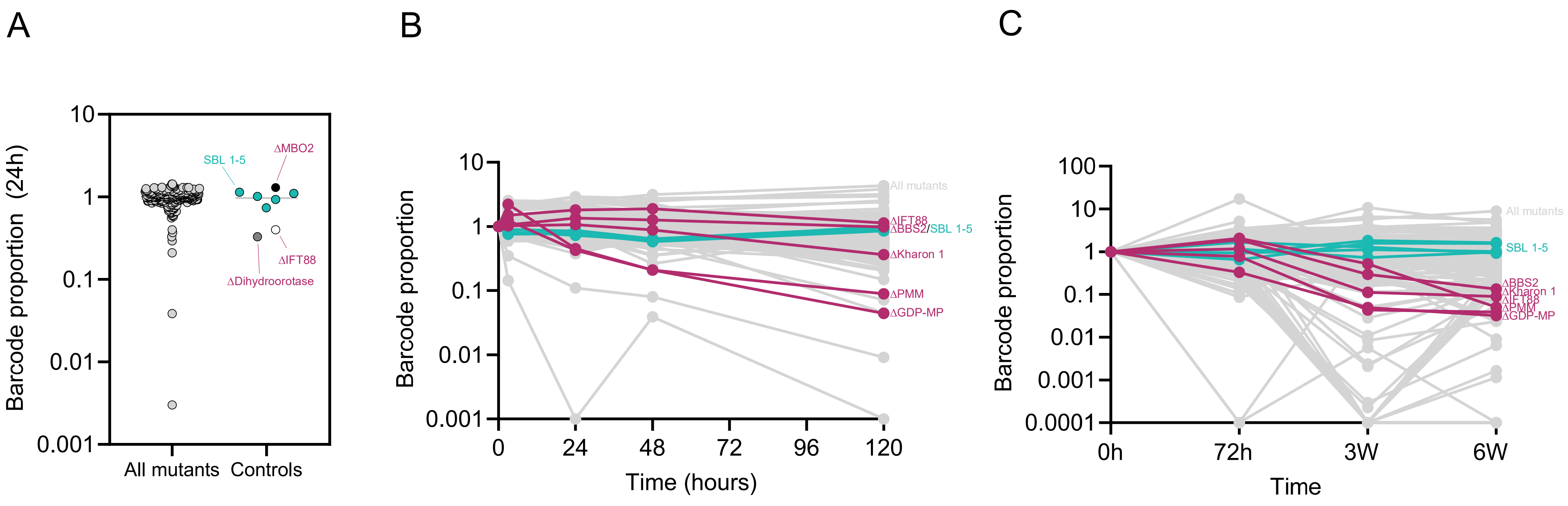

Supplementary Figure 6. Barcode proportions over time for all experiments.

(A) Barcode proportions in promastigote cultures at 24 h (normalised to 0 h). (B) Trajectories of barcode proportions at 24, 48, 72, 96 and 120 h (normalised to 0 h) in samples from infected macrophages. (C) Trajectories of barcode proportions at 72 h, 3 and 6 weeks (normalised to 0 h) in samples taken from infected mice. Turquoise, parental controls (SBL1-5); magenta, control knockout mutants; grey, all other transporter mutants. Each dot represents the value for one barcoded cell line at the respective time point. The source data are provided in Supplementary Data 4.

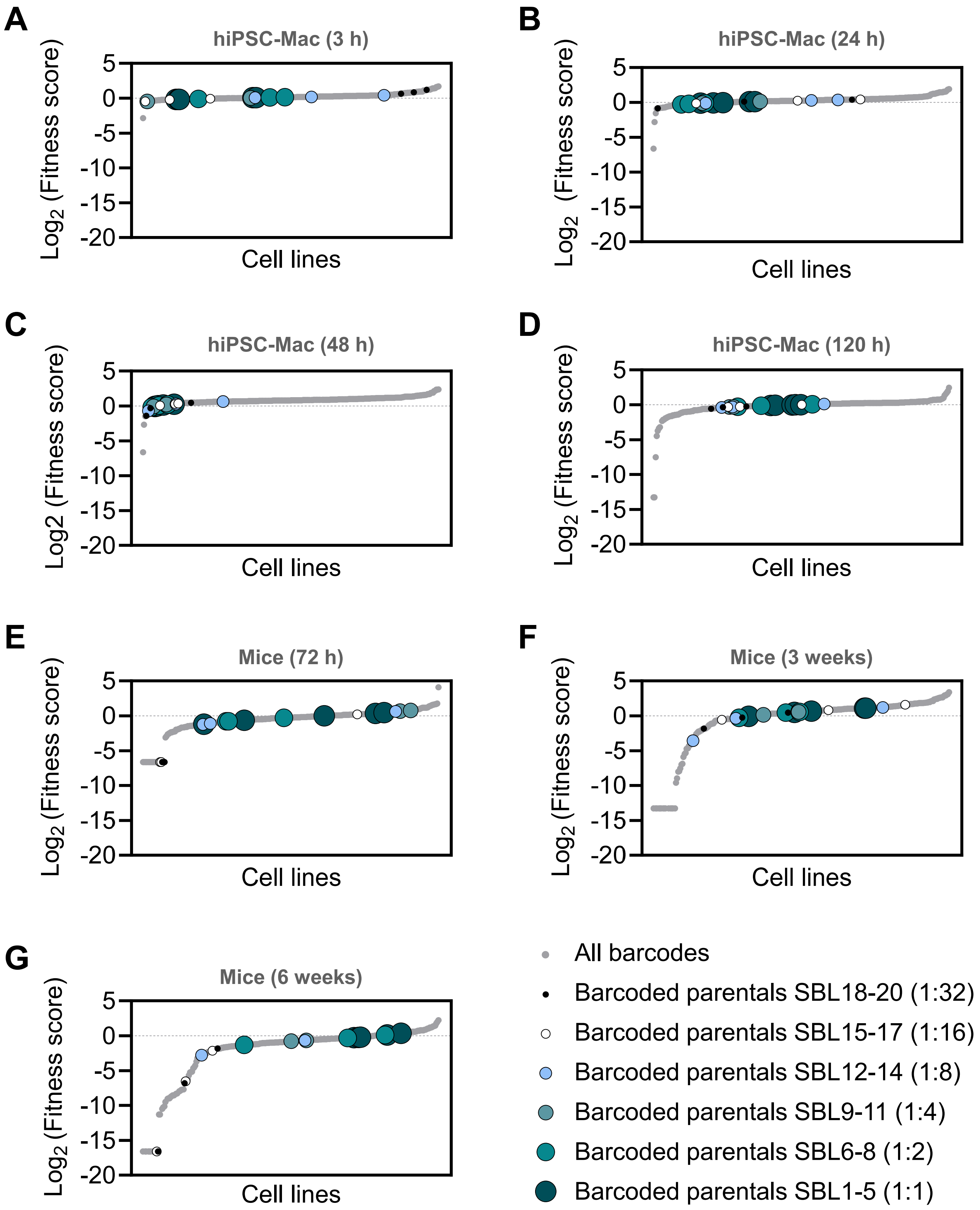

Supplementary Figure 7. Fitness scores of SBL titration.

TransLeish cell lines ranked in order of their fitness scores in hiPSC-Mac (A-D) or in mice (E-G). Mutant cell lines are depicted as small grey dots, the parental control cell lines are shown as larger dots, coloured according to the dilution at which they were added to the pool. The source data are provided in Supplementary Data 4.

Source Images for Supplementary Figure 5

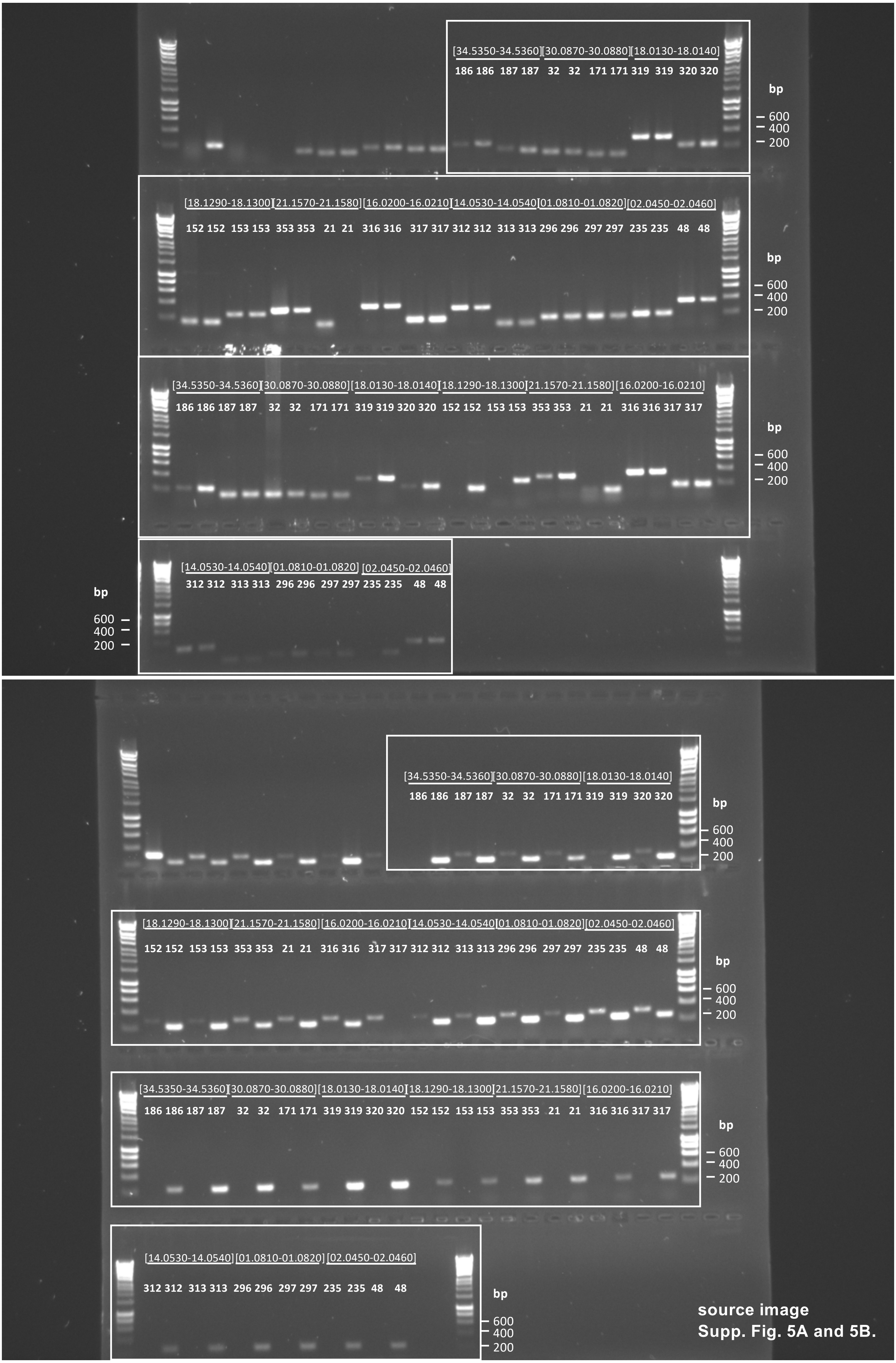

source image
Supp. Fig. 5A and 5B.

Uncropped source image for Supp. Fig. 5A and 5B. The white boxes outline the cropped areas. The unlabelled lanes contain samples unrelated to the results reported in this paper.

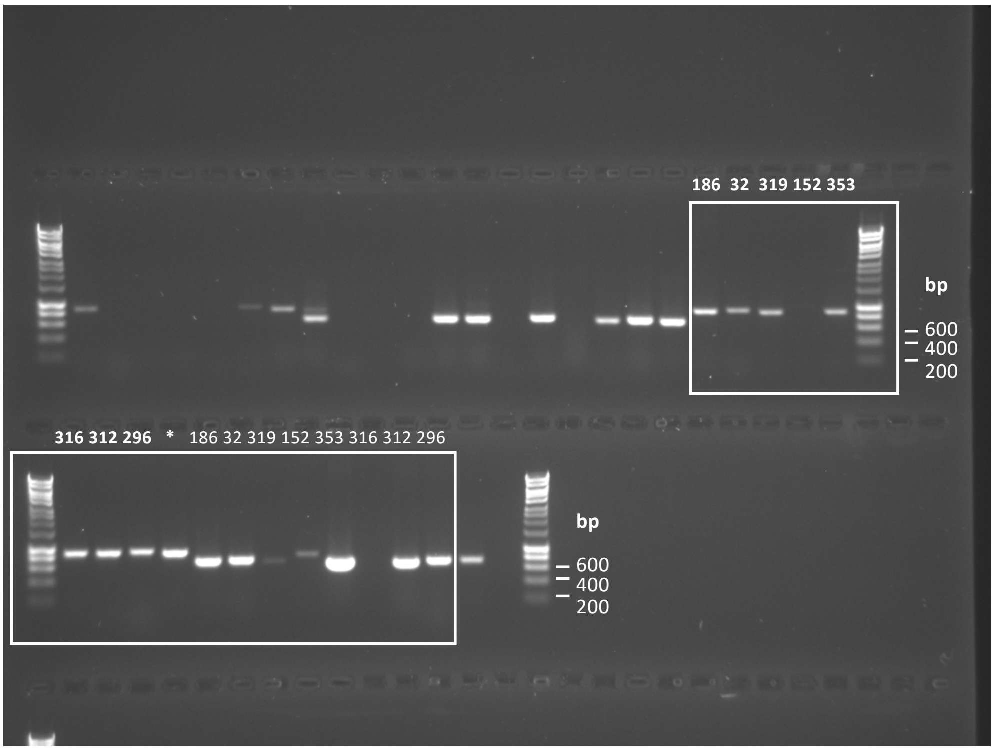

Uncropped source image for Supp. Fig. 5C. The white boxes outline the cropped areas. The unlabelled lanes contain samples unrelated to the results reported in this paper.

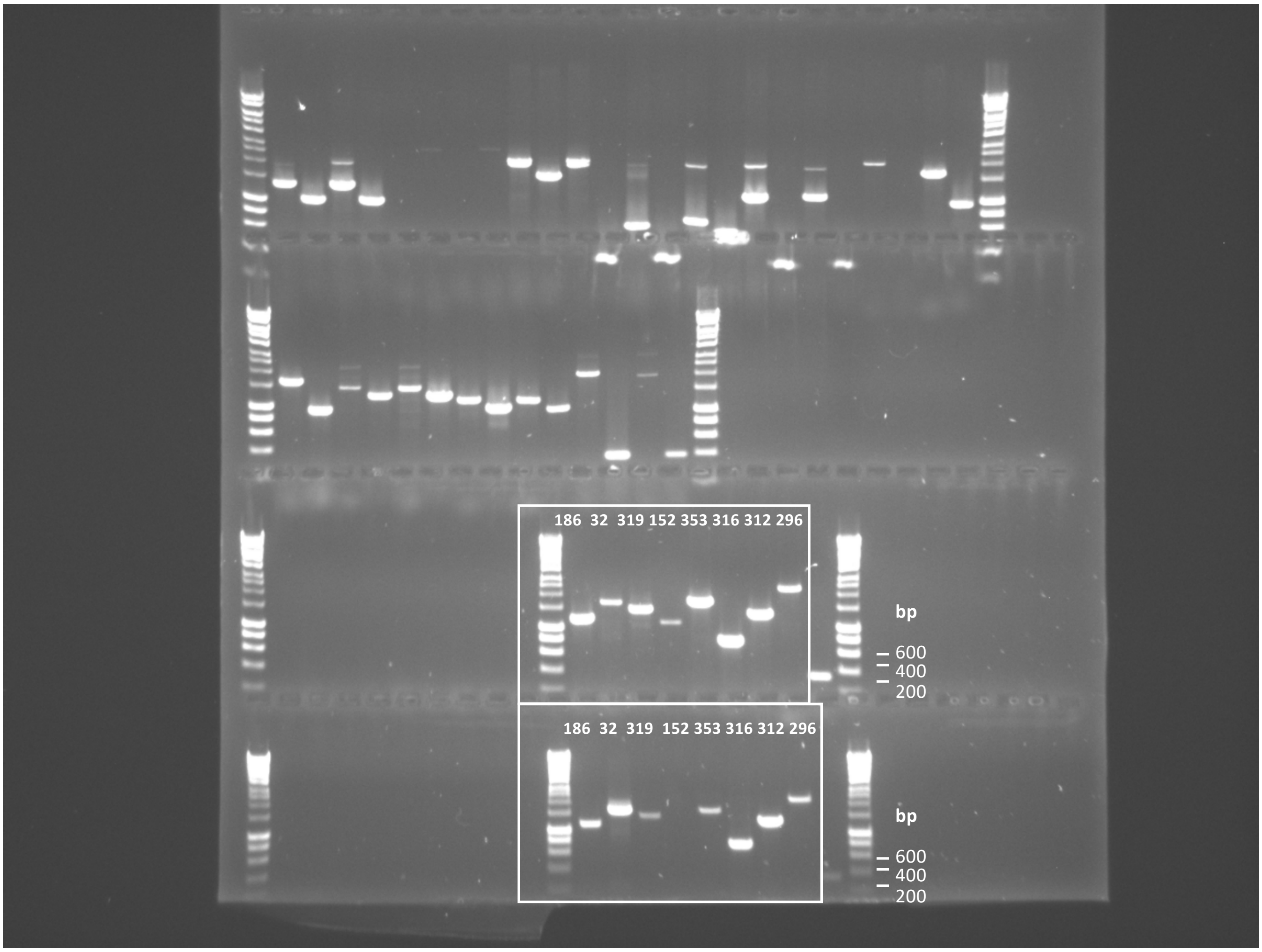

Uncropped source image for Supp. Fig. 5D. The white boxes outline the cropped areas. The unlabelled lanes contain samples unrelated to the results reported in this paper.

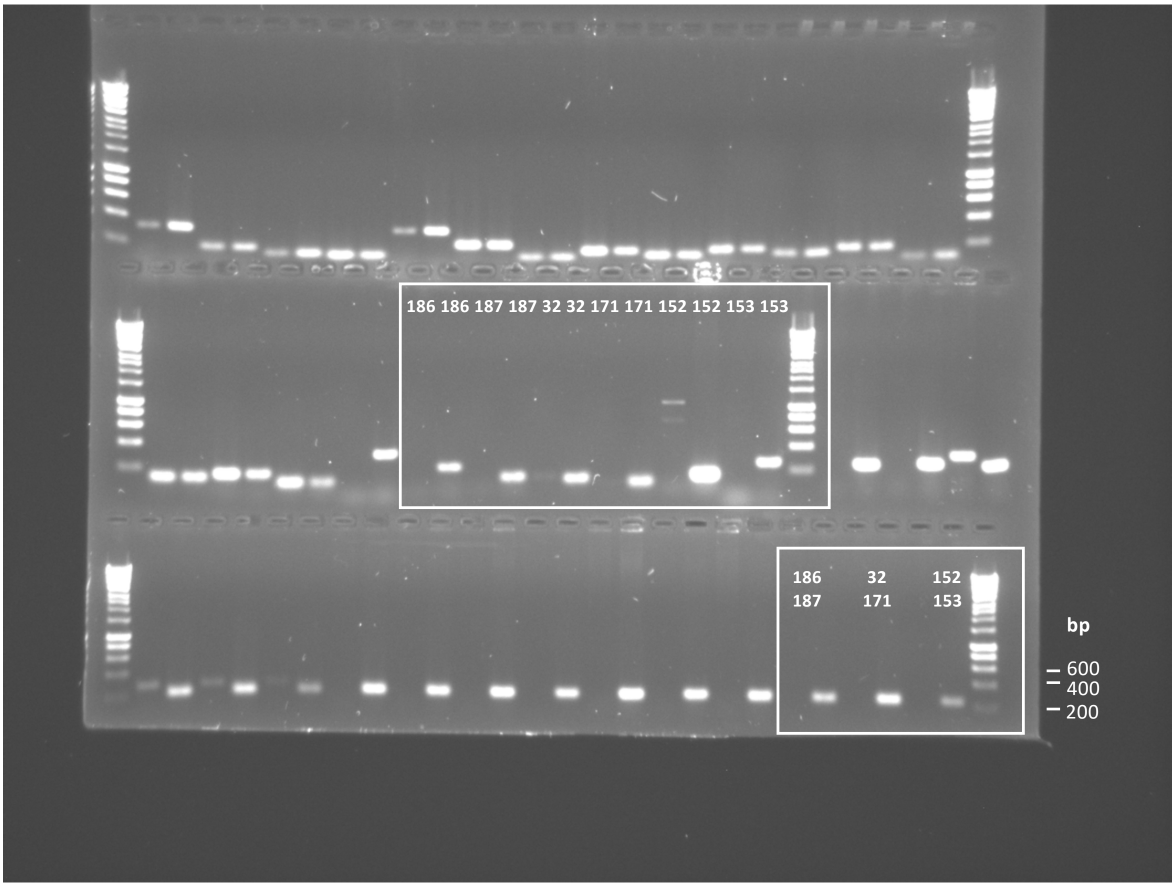

Uncropped source image for Supp. Fig. 5E and 5F. The white boxes outline the cropped areas. The unlabelled lanes contain samples unrelated to the results reported in this paper.

References for Supplementary Data 1 - TransLeishDB

1 Benaim, G., Garcia-Marchan, Y., Reyes, C., Uzcanga, G. & Figarella, K. Identification of a sphingosine-sensitive Ca2+ channel in the plasma membrane of *Leishmania mexicana*. *Biochemical and Biophysical Research Communications* **430**, 1091-1096, doi:10.1016/j.bbrc.2012.12.033 (2013).

2 Rodriguez-Duran, J., Pinto-Martinez, A., Castillo, C. & Benaim, G. Identification and electrophysiological properties of a sphingosine-dependent plasma membrane Ca(2+) channel in *Trypanosoma cruzi*. *The FEBS Journal* **286**, 3909-3925, doi:10.1111/febs.14947 (2019).

3 Steinmann, M. E., Gonzalez-Salgado, A., Bütikofer, P., Mäser, P. & Sigel, E. A heteromeric potassium channel involved in the modulation of the plasma membrane potential is essential for the survival of African trypanosomes. *The FASEB Journal : official publication of the Federation of American Societies for Experimental Biology* **29**, 3228-3237, doi:10.1096/fj.15-271353 (2015).

4 Jimenez, V. & Docampo, R. Molecular and electrophysiological characterization of a novel cation channel of *Trypanosoma cruzi*. *PLoS Pathogens* **8**, e1002750, doi:10.1371/journal.ppat.1002750 (2012).

5 Dave, N. *et al.* A novel mechanosensitive channel controls osmoregulation, differentiation, and infectivity in *Trypanosoma cruzi*. *eLife* **10**, doi:10.7554/eLife.67449 (2021).

6 Huang, G., Bartlett, P. J., Thomas, A. P., Moreno, S. N. & Docampo, R. Acidocalcisomes of Trypanosoma brucei have an inositol 1,4,5-trisphosphate receptor that is required for growth and infectivity. *Proceedings of the National Academy of Sciences of the United States of America* **110**, 1887-1892, doi:10.1073/pnas.1216955110 (2013).

7 Zhu, Y., Davis, A., Smith, B. J., Curtis, J. & Handman, E. Leishmania major CorA-like magnesium transporters play a critical role in parasite development and virulence. *International Journal for Parasitology* **39**, 713-723, doi:10.1016/j.ijpara.2008.11.010 (2009).

8 Rojas, F. *et al.* Oligopeptide Signaling through TbGPR89 Drives Trypanosome Quorum Sensing. *Cell* **176**, 306-317 e316, doi:10.1016/j.cell.2018.10.041 (2019).

9 Docampo, R. & Huang, G. Calcium signaling in trypanosomatid parasites. *Cell Calcium* **57**, 194-202, doi:10.1016/j.ceca.2014.10.015 (2015).

10 Prole, D. L. & Taylor, C. W. Identification and analysis of putative homologues of mechanosensitive channels in pathogenic protozoa. *PloS One* **8**, e66068, doi:10.1371/journal.pone.0066068 (2013).

11 Huang, G. & Docampo, R. The Mitochondrial Ca(2+) Uniporter Complex (MCUC) of *Trypanosoma brucei* Is a Hetero-oligomer That Contains Novel Subunits Essential for Ca(2+) Uptake. *mBio* **9**, doi:10.1128/mBio.01700-18 (2018).

12 Chiurillo, M. A., Lander, N., Bertolini, M. S., Vercesi, A. E. & Docampo, R. Functional analysis and importance for host cell infection of the Ca(2+)-conducting subunits of the mitochondrial calcium uniporter of *Trypanosoma cruzi*. *Molecular Biology of the Cell* **30**, 1676-1690, doi:10.1091/mbc.E19-03-0152 (2019).

13 Plourde, M. *et al.* Generation of an aquaglyceroporin AQP1 null mutant in *Leishmania major*. *Molecular and Biochemical Parasitology* **201**, 108-111, doi:10.1016/j.molbiopara.2015.07.003 (2015).

14 Singha, U. K., Sharma, S. & Chaudhuri, M. Downregulation of mitochondrial porin inhibits cell growth and alters respiratory phenotype in *Trypanosoma brucei*. *Eukaryotic Cell* **8**, 1418-1428, doi:10.1128/EC.00132-09 (2009).

15 Cabello-Donayre, M. *et al.* *Leishmania* heme uptake involves LmFLVCRb, a novel porphyrin transporter essential for the parasite. *Cell Mol Life Sci* **77**, 1827-1845, doi:10.1007/s00018-019-03258-3 (2020).
16 Burchmore, R. J. *et al.* Genetic characterization of glucose transporter function in *Leishmania mexicana*. *Proceedings of the National Academy of Sciences of the United States of America* **100**, 3901-3906, doi:10.1073/pnas.0630165100 (2003).
17 Feng, X. *et al.* Amplification of an alternate transporter gene suppresses the avirulent phenotype of glucose transporter null mutants in *Leishmania mexicana*. *Molecular Microbiology* **71**, 369-381, doi:10.1111/j.1365-2958.2008.06531.x (2009).
18 Balcazar, D. E. *et al.* The superfamily keeps growing: Identification in trypanosomatids of RibJ, the first riboflavin transporter family in protists. *PLoS Negl Trop Dis* **11**, e0005513, doi:10.1371/journal.pntd.0005513 (2017).
19 Dean, S., Marchetti, R., Kirk, K. & Matthews, K. R. A surface transporter family conveys the trypanosome differentiation signal. *Nature* **459**, 213-217, doi:10.1038/nature07997 (2009).
20 Laranjeira-Silva, M. F. *et al.* A MFS-like plasma membrane transporter required for *Leishmania* virulence protects the parasites from iron toxicity. *PLoS Pathogens* **14**, e1007140, doi:10.1371/journal.ppat.1007140 (2018).
21 Drew, M. E. *et al.* Functional expression of a myo-inositol/H+ symporter from *Leishmania donovani*. *Molecular and Cellular Biology* **15**, 5508-5515, doi:10.1128/MCB.15.10.5508 (1995).
22 Sanchez, M. A. Molecular identification and characterization of an essential pyruvate transporter from *Trypanosoma brucei*. *The Journal of Biological Chemistry* **288**, 14428-14437, doi:10.1074/jbc.M113.473157 (2013).
23 Stafkova, J. *et al.* Mitochondrial pyruvate carrier in *Trypanosoma brucei*. *Molecular Microbiology* **100**, 442-456, doi:10.1111/mmi.13325 (2016).
24 Ramakrishnan, S., Unger, L. M., Baptista, R. P., Cruz-Bustos, T. & Docampo, R. Deletion of a Golgi protein in *Trypanosoma cruzi* reveals a critical role for Mn2+ in protein glycosylation needed for host cell invasion and intracellular replication. *PLoS Pathogens* **17**, e1009399, doi:10.1371/journal.ppat.1009399 (2021).
25 Huynh, C. *et al.* Heme uptake by *Leishmania amazonensis* is mediated by the transmembrane protein LHR1. *PLoS Pathogens* **8**, e1002795, doi:10.1371/journal.ppat.1002795 (2012).
26 Osorio-Mendez, J. F., Tellez, G. A., Zapata-Lopez, D., Echeverry, S. & Castano, J. C. Sequence analysis of SWEET transporters from trypanosomatids and evaluation of its expression in *Trypanosoma cruzi*. *Experimental Parasitology* **248**, 108496, doi:10.1016/j.exppara.2023.108496 (2023).
27 Jackson, A. P. Origins of amino acid transporter loci in trypanosomatid parasites. *BMC Evolutionary Biology* **7**, 26, doi:10.1186/1471-2148-7-26 (2007).
28 Shaked-Mishan, P. *et al.* A novel high-affinity arginine transporter from the human parasitic protozoan *Leishmania donovani*. *Molecular Microbiology* **60**, 30-38, doi:10.1111/j.1365-2958.2006.05060.x (2006).
29 Inbar, E., Schlisselberg, D., Suter Grotemeyer, M., Rentsch, D. & Zilberstein, D. A versatile proline/alanine transporter in the unicellular pathogen *Leishmania donovani* regulates amino acid homoeostasis and osmotic stress responses. *The Biochemical Journal* **449**, 555-566, doi:10.1042/BJ20121262 (2013).
30 Roy, G., Bhattacharya, A., Leprohon, P. & Ouellette, M. Decreased glutamate transport in acivicin resistant *Leishmania tarentolae*. *PLoS Negl Trop Dis* **15**, e0010046, doi:10.1371/journal.pntd.0010046 (2021).
31 Inbar, E. *et al.* Lysine transporters in human trypanosomatid pathogens. *Amino Acids* **42**, 347-360, doi:10.1007/s00726-010-0812-z (2012).

32 Russo-Abrahao, T. *et al.* Transport of inorganic phosphate in *Leishmania infantum* and compensatory regulation at low inorganic phosphate concentration. *Biochimica et Biophysica Acta* **1830**, 2683-2689 (2013).

33 Mittra, B. *et al.* A Trypanosomatid Iron Transporter that Regulates Mitochondrial Function Is Required for *Leishmania amazonensis* Virulence. *PLoS Pathogens* **12**, e1005340, doi:10.1371/journal.ppat.1005340 (2016).

34 Colasante, C., Pena Diaz, P., Clayton, C. & Voncken, F. Mitochondrial carrier family inventory of Trypanosoma brucei brucei: Identification, expression and subcellular localisation. *Molecular and Biochemical Parasitology* **167**, 104-117, doi:10.1016/j.molbiopara.2009.05.004 (2009).

35 Rotureau, B., Morales, M. A., Bastin, P. & Späth, G. F. The flagellum-MAP kinase connection in Trypanosomatids: a key sensory role in parasite signaling and development? *Cellular Microbiology*, doi:CMI1295 [pii] 10.1111/j.1462-5822.2009.01295.x (2009).

36 Hasne, M. P. & Ullman, B. Identification and characterization of a polyamine permease from the protozoan parasite *Leishmania major*. *The Journal of Biological Chemistry* **280**, 15188-15194, doi:10.1074/jbc.M411331200 (2005).

37 Mosimann, M. *et al.* A Trk/HKT-type K+ transporter from *Trypanosoma brucei*. *Eukaryotic Cell* **9**, 539-546, doi:10.1128/EC.00314-09 (2010).

38 Steinmann, M. E. *et al.* Identification and characterization of the three members of the CLC family of anion transport proteins in *Trypanosoma brucei*. *PloS One* **12**, e0188219, doi:10.1371/journal.pone.0188219 (2017).

39 Huynh, C., Sacks, D. L. & Andrews, N. W. A *Leishmania amazonensis* ZIP family iron transporter is essential for parasite replication within macrophage phagolysosomes. *The Journal of Experimental Medicine* **203**, 2363-2375, doi:jem.20060559 [pii] 10.1084/jem.20060559 (2006).

40 Carvalho, S. *et al.* LiZIP3 is a cellular zinc transporter that mediates the tightly regulated import of zinc in *Leishmania infantum* parasites. *Molecular Microbiology* **96**, 581-595, doi:10.1111/mmi.12957 (2015).

41 Carter, N. S. *et al.* Cloning of a novel inosine-guanosine transporter gene from *Leishmania donovani* by functional rescue of a transport-deficient mutant. *The Journal of Biological Chemistry* **275**, 20935-20941, doi:10.1074/jbc.M002418200 (2000).

42 Aldfer, M. M. *et al.* Nucleoside Transport and Nucleobase Uptake Null Mutants in *Leishmania mexicana* for the Routine Expression and Characterization of Purine and Pyrimidine Transporters. *Int J Mol Sci* **23**, doi:10.3390/ijms23158139 (2022).

43 Ishemgulova, A. *et al.* CRISPR/Cas9 in *Leishmania mexicana*: A case study of LmxBTN1. *PloS One* **13**, e0192723, doi:10.1371/journal.pone.0192723 (2018).

44 Al-Salabi, M. I., Wallace, L. J. & De Koning, H. P. A *Leishmania major* nucleobase transporter responsible for allopurinol uptake is a functional homolog of the Trypanosoma brucei H2 transporter. *Mol Pharmacol* **63**, 814-820, doi:10.1124/mol.63.4.814 (2003).

45 Ortiz, D. *et al.* Molecular genetic analysis of purine nucleobase transport in *Leishmania major*. *Molecular Microbiology* **64**, 1228-1243, doi:10.1111/j.1365-2958.2007.05730.x (2007).

46 Sanchez, M. A., Tryon, R., Pierce, S., Vasudevan, G. & Landfear, S. M. Functional expression and characterization of a purine nucleobase transporter gene from *Leishmania major*. *Mol Membr Biol* **21**, 11-18, doi:10.1080/0968768031000140845 (2004).

47 Ilg, T., Demar, M. & Harbecke, D. Phosphoglycan repeat-deficient *Leishmania mexicana* parasites remain infectious to macrophages and mice. *The Journal of Biological Chemistry* **276**, 4988-4997, doi:10.1074/jbc.M008030200 (2001).

48 Späth, G. F., Garraway, L. A., Turco, S. J. & Beverley, S. M. The role(s) of lipophosphoglycan (LPG) in the establishment of *Leishmania major* infections in mammalian hosts. *Proceedings of the National Academy of Sciences of the United States of America* **100**, 9536-9541, doi:10.1073/pnas.1530604100 1530604100 [pii] (2003).

49 Ma, D., Russell, D. G., Beverley, S. M. & Turco, S. J. Golgi GDP-mannose uptake requires Leishmania LPG2. A member of a eukaryotic family of putative nucleotide-sugar transporters. *The Journal of Biological Chemistry* **272**, 3799-3805 (1997).

50 Capul, A. A., Barron, T., Dobson, D. E., Turco, S. J. & Beverley, S. M. Two functionally divergent UDP-Gal nucleotide sugar transporters participate in phosphoglycan synthesis in *Leishmania major*. *The Journal of Biological Chemistry* **282**, 14006-14017, doi:10.1074/jbc.M610869200 (2007).

51 Ouameur, A. A., Girard, I., Legare, D. & Ouellette, M. Functional analysis and complex gene rearrangements of the folate/biopterin transporter (FBT) gene family in the protozoan parasite *Leishmania*. *Molecular and Biochemical Parasitology* **162**, 155-164, doi:10.1016/j.molbiopara.2008.08.007 (2008).

52 Richard, D., Kundig, C. & Ouellette, M. A new type of high affinity folic acid transporter in the protozoan parasite *Leishmania* and deletion of its gene in methotrexate-resistant cells. *The Journal of Biological Chemistry* **277**, 29460-29467, doi:10.1074/jbc.M204796200 (2002).

53 Papadopoulou, B. *et al.* Reduced infectivity of a *Leishmania donovani* biopterin transporter genetic mutant and its use as an attenuated strain for vaccination. *Infect Immun* **70**, 62-68, doi:10.1128/IAI.70.1.62-68.2002 (2002).

54 Yernaux, C., Fransen, M., Brees, C., Lorenzen, S. & Michels, P. A. *Trypanosoma brucei* glycosomal ABC transporters: identification and membrane targeting. *Mol Membr Biol* **23**, 157-172, doi:10.1080/09687860500460124 (2006).

55 Manzano, J. I. *et al.* Leishmania LABCG1 and LABCG2 transporters are involved in virulence and oxidative stress: functional linkage with autophagy. *Parasites & Vectors* **10**, 267, doi:10.1186/s13071-017-2198-1 (2017).

56 Campos-Salinas, J. *et al.* A new ATP-binding cassette protein is involved in intracellular haem trafficking in *Leishmania*. *Molecular Microbiology* **79**, 1430-1444, doi:10.1111/j.1365-2958.2010.07531.x (2011).

57 Martinez-Garcia, M. *et al.* LmABCB3, an atypical mitochondrial ABC transporter essential for *Leishmania major* virulence, acts in heme and cytosolic iron/sulfur clusters biogenesis. *Parasites & Vectors* **9**, 7, doi:10.1186/s13071-015-1284-5 (2016).

58 Hendrickson, N. *et al.* Molecular characterization of the ldmdr1 multidrug resistance gene from *Leishmania donovani*. *Molecular and Biochemical Parasitology* **60**, 53-64, doi:10.1016/0166-6851(93)90028-v (1993).

59 Leprohon, P., Legare, D., Girard, I., Papadopoulou, B. & Ouellette, M. Modulation of Leishmania ABC protein gene expression through life stages and among drug-resistant parasites. *Eukaryotic Cell* **5**, 1713-1725, doi:10.1128/EC.00152-06 (2006).

60 Coelho, A. C., Beverley, S. M. & Cotrim, P. C. Functional genetic identification of PRP1, an ABC transporter superfamily member conferring pentamidine resistance in *Leishmania major*. *Molecular and Biochemical Parasitology* **130**, 83-90, doi:10.1016/s0166-6851(03)00162-2 (2003).

61 Arcari, T., Manzano, J. I. & Gamarro, F. ABCI3 Is a New Mitochondrial ABC Transporter from *Leishmania major* Involved in Susceptibility to Antimonials and Infectivity. *Antimicrobial Agents and Chemotherapy* **61**, doi:10.1128/AAC.01115-17 (2017).

62 do Monte-Neto, R. L. *et al.* Gene expression profiling and molecular characterization of antimony resistance in *Leishmania amazonensis*. *PLoS Negl Trop Dis* **5**, e1167, doi:10.1371/journal.pntd.0001167 (2011).

63 Araujo-Santos, J. M., Parodi-Talice, A., Castanys, S. & Gamarro, F. The overexpression of an intracellular ABCA-like transporter alters phospholipid trafficking in *Leishmania*. *Biochemical and Biophysical Research Communications* **330**, 349-355, doi:10.1016/j.bbrc.2005.02.176 (2005).

64 Parodi-Talice, A. *et al.* The overexpression of a new ABC transporter in *Leishmania* is related to phospholipid trafficking and reduced infectivity. *Biochimica et Biophysica Acta* **1612**, 195-207, doi:10.1016/s0005-2736(03)00131-7 (2003).

65 Castanys-Munoz, E., Perez-Victoria, J. M., Gamarro, F. & Castanys, S. Characterization of an ABCG-like transporter from the protozoan parasite *Leishmania* with a role in drug resistance and transbilayer lipid movement. *Antimicrobial Agents and Chemotherapy* **52**, 3573-3579, doi:10.1128/AAC.00587-08 (2008).

66 Zhang, W. W. & Matlashewski, G. Deletion of an ATP-binding cassette protein subfamily C transporter in *Leishmania donovani* results in increased virulence. *Molecular and Biochemical Parasitology* **185**, 165-169, doi:10.1016/j.molbiopara.2012.07.006 (2012).

67 Rodrigues, C. O., Scott, D. A. & Docampo, R. Characterization of a vacuolar pyrophosphatase in Trypanosoma brucei and its localization to acidocalcisomes. *Molecular and Cellular Biology* **19**, 7712-7723, doi:10.1128/MCB.19.11.7712 (1999).

68 Meade, J. C. P-type transport ATPases in *Leishmania* and *Trypanosoma*. *Parasite* **26**, 69, doi:10.1051/parasite/2019069 (2019).

69 Baker, N. *et al.* Vacuolar ATPase depletion affects mitochondrial ATPase function, kinetoplast dependency, and drug sensitivity in trypanosomes. *Proceedings of the National Academy of Sciences of the United States of America* **112**, 9112-9117, doi:10.1073/pnas.1505411112 (2015).

70 Bertolini, M. S., Chiurillo, M. A., Lander, N., Vercesi, A. E. & Docampo, R. MICU1 and MICU2 Play an Essential Role in Mitochondrial Ca(2+) Uptake, Growth, and Infectivity of the Human Pathogen *Trypanosoma cruzi*. *mBio* **10**, doi:10.1128/mBio.00348-19 (2019).
