# Supplementary material for: TransLeish: Identification of membrane transporters essential for survival of intracellular Leishmania parasites in a systematic gene deletion screen
Source: Nat Commun. 2025 Jan 2;16:299. doi: 10.1038/s41467-024-55538-7 (PMC11696137; doi:10.1038/s41467-024-55538-7)
Supplement: Supplementary file 2 — Description Of Additional Supplementary File [file 41467_2024_55538_MOESM2_ESM.pdf]

Description of Additional supplementary files

Supplementary Data 1.

Description: Information about genes targeted in the knockout screen and the resulting mutant cell lines

Supplementary Data 2.

Description: Results of diagnostic PCRs for knockout validations for all mutant cell lines

Supplementary Data 3.

Description: Tab “Table 3A”: ID of genes in tandem arrays. Tab “Table 3B”: Arrays considered for deletion. Tab “Table 3C”: Diagnostic PCR for Blasticidin resistant mutants. Tab “Table 3D”: Diagnostic PCR of Puromycin resistant mutants. Tab “Table 3E”: Diagnostic PCR of Blasticidin and Puromycin resistant mutants. Tab “Table 3F” - BlastP % protein sequence ID.

Supplementary Data 4.

Description: Fitness scores, p-values from Mann-Whitney U test, raw read counts and summary of reads for each timepoint from promastigotes grown in vitro, hiPSC-Mac infections (Mac 3h, Mac 24h, Mac 48h, Mac 120h) and infection of mice (FP 72h, FP 3w and FP 6w).

Supplementary Data 5.

Description: Barcode and primer sequences used for the generation of mutant cell lines and diagnostic PCRs.
