# Supplementary material for: TransLeish: Identification of membrane transporters essential for survival of intracellular Leishmania parasites in a systematic gene deletion screen
Source: Nat Commun. 2025 Jan 2;16:299. doi: 10.1038/s41467-024-55538-7 (PMC11696137; doi:10.1038/s41467-024-55538-7)
Supplement: Supplementary file 8 — Reporting summary [file 41467_2024_55538_MOESM8_ESM.pdf]

nature portfolio

Corresponding author(s): E. Gluenz

Last updated by author(s): 13/12/24

Reporting Summary

Nature Portfolio wishes to improve the reproducibility of the work that we publish. This form provides structure for consistency and transparency in reporting. For further information on Nature Portfolio policies, see our Editorial Policies and the Editorial Policy Checklist.

Statistics

For all statistical analyses, confirm that the following items are present in the figure legend, table legend, main text, or Methods section.

n/a	Confirmed	
☐	☒	The exact sample size (*n*) for each experimental group/condition, given as a discrete number and unit of measurement
☐	☒	A statement on whether measurements were taken from distinct samples or whether the same sample was measured repeatedly
☐	☒	The statistical test(s) used AND whether they are one- or two-sided<br>*Only common tests should be described solely by name; describe more complex techniques in the Methods section.*
☒	☐	A description of all covariates tested
☒	☐	A description of any assumptions or corrections, such as tests of normality and adjustment for multiple comparisons
☐	☒	A full description of the statistical parameters including central tendency (e.g. means) or other basic estimates (e.g. regression coefficient) AND variation (e.g. standard deviation) or associated estimates of uncertainty (e.g. confidence intervals)
☐	☒	For null hypothesis testing, the test statistic (e.g. *F*, *t*, *r*) with confidence intervals, effect sizes, degrees of freedom and *P* value noted<br>*Give P values as exact values whenever suitable.*
☒	☐	For Bayesian analysis, information on the choice of priors and Markov chain Monte Carlo settings
☒	☐	For hierarchical and complex designs, identification of the appropriate level for tests and full reporting of outcomes
☒	☐	Estimates of effect sizes (e.g. Cohen's *d*, Pearson's *r*), indicating how they were calculated

Our web collection on statistics for biologists contains articles on many of the points above.

Software and code

Policy information about availability of computer code

Data collection	No custom software was used.
Data analysis	Cell line barcode sequence counts were extracted using LeishFASTQ version 1.0 (available from https://zenodo.org/records/14288099). Custom code for the statistical analysis of cell line fitness was written in phyton 3.8.10 using pandas 2.0.3, scipy 1.8.0, numpy 1.21.3, matplotlib 3.7.1, IPython 7.29.0 and notebook 6.4.5 and is available on GitHub (https//github.com/ulido/TransLeish1/tree/paper_submission). All graph plots presented in the manuscript figures were produced using GraphPad Prism v10.2.3.

For manuscripts utilizing custom algorithms or software that are central to the research but not yet described in published literature, software must be made available to editors and reviewers. We strongly encourage code deposition in a community repository (e.g. GitHub). See the Nature Portfolio guidelines for submitting code & software for further information.

Data

Policy information about availability of data

All manuscripts must include a data availability statement. This statement should provide the following information, where applicable:

- Accession codes, unique identifiers, or web links for publicly available datasets
- A description of any restrictions on data availability
- For clinical datasets or third party data, please ensure that the statement adheres to our policy

All datasets generated and analysed are available in the publication including supplementary files. Whole genome sequencing and bar-seq raw data have been deposited at the European Nucleotide Archive under the study accession number: PRJEB76744.

Research involving human participants, their data, or biological material

Policy information about studies with human participants or human data. See also policy information about sex, gender (identity/presentation), and sexual orientation and race, ethnicity and racism.

Reporting on sex and gender	Not applicable
Reporting on race, ethnicity, or other socially relevant groupings	Not applicable
Population characteristics	Not applicable
Recruitment	Not applicable
Ethics oversight	Three human induced pluripotent stem cell (hiPSC) lines were used in this study, which have been published previously (Dafinca et al., 2016; Fernandes et al., 2016; Haenseler et al., 2017) and are deposited in EBiSC (STBCi026-A, STBCi044-A, STBCi063-A).<br>Ethical approval for the derivation and use of these cells was obtained (Ethics Committee: National Health Service, Health Research Authority, NRES Committee South Central, Berkshire, UK, REC 10/H0505/71) with written informed consent by donors that specifically stated that their skin biopsies would be used for the derivation of pluripotent stem cell lines, and sharing of the cells with other researchers beyond the original deriving research team. This includes differentiation to cell types including immune cells, as in this manuscript.

Note that full information on the approval of the study protocol must also be provided in the manuscript.

Field-specific reporting

Please select the one below that is the best fit for your research. If you are not sure, read the appropriate sections before making your selection.

☒ Life sciences ☐ Behavioural & social sciences ☐ Ecological, evolutionary & environmental sciences

For a reference copy of the document with all sections, see nature.com/documents/nr-reporting-summary-flat.pdf

Life sciences study design

All studies must disclose on these points even when the disclosure is negative.

Sample size	The number of barcoded cell lines included in each pool and the number of replicates for each time point were based on existing datasets from similar experiments (e.g. PMID: 33623024) taking into account expected variation and ethical considerations. Experiments on animals used six mice per time point, experiments using cultured parasites used three replicates per experiment. Bar-seq analysis was based on principles described in PMID: 24192834.
Data exclusions	For mouse and macrophage infections, two sets of mutants from separate studies (transporter mutants and flagellum mutants) were combined in a "masterpool" to reduce the number of animals used in these studies. The bar-seq analysis of barcode proportions and statistical significance of any change was done jointly for all mutants included in the masterpool (data deposited in ENA and Supplementary Data files). This paper reports the detailed analysis of the transporter mutants only. A separate paper will report the detailed analysis of the 53 non-transporter mutants.
Replication	Where applicable, experimental replicates are stated in the figure legends.
Randomization	Animals were selected randomly for each group.
Blinding	Blinding was not possible in the animal models.

Reporting for specific materials, systems and methods

We require information from authors about some types of materials, experimental systems and methods used in many studies. Here, indicate whether each material, system or method listed is relevant to your study. If you are not sure if a list item applies to your research, read the appropriate section before selecting a response.

Materials & experimental systems

n/a	Involved in the study	
☒	☐	Antibodies
☐	☒	Eukaryotic cell lines
☒	☐	Palaeontology and archaeology
☐	☒	Animals and other organisms
☒	☐	Clinical data
☒	☐	Dual use research of concern
☒	☐	Plants

Methods

n/a	Involved in the study	
☒	☐	ChIP-seq
☒	☐	Flow cytometry
☒	☐	MRI-based neuroimaging

Eukaryotic cell lines

Policy information about cell lines and Sex and Gender in Research

Cell line source(s)	Leishmania mexicana (MNYC/BZ/62/M379) expressing T7 RNA Polymerase reported in PMID: 28573017 and PMID: 36874584
Authentication	The parasite cell line was authenticated via testing for expected phenotypes in vivo and in vitro and by whole genome sequencing.
Mycoplasma contamination	Not tested
Commonly misidentified lines (See ICLAC register)	No commonly misidentified lines were used in this study.

Animals and other research organisms

Policy information about studies involving animals; ARRIVE guidelines recommended for reporting animal research, and Sex and Gender in Research

Laboratory animals	As partially described in the Methods, female BALB/c mice (ref.: 028-BalB/cAnNCrl) of 8 weeks, purchased from Charles River Laboratories were socially housed in IVC caging, 19-23 C with humidity levels varying between 45-65% and a 12 hour light/dark cycle, from 7:30 am to 7:30 pm. The animals were maintained in the pathogen-free facility at the University of York and were only used for this experiment.
Wild animals	The study did not use animals collected from the wild.
Reporting on sex	Mouse models were always female. The findings are assumed to apply to both sexes. Female mice were chosen because males are aggressive and often fight and get injured, which interferes with data collection.
Field-collected samples	The study did not use animals collected from the wild.
Ethics oversight	All experiments were conducted according to the Animals (Scientific Procedures) Act of 1986, United Kingdom, and had approval from the University of York, Animal and Ethical Review Body (AWERB) committee (protocol number PP1651724).

Note that full information on the approval of the study protocol must also be provided in the manuscript.

Plants

Seed stocks	Not applicable
Novel plant genotypes	Not applicable
Authentication	Not applicable
